# Examining systemic differences in mortality after hip repair: a comparative analysis of 30- and 180-day adjusted mortality rates in five health systems

**DOI:** 10.1093/eurpub/ckaf074

**Published:** 2025-06-26

**Authors:** Francisco Estupiñán-Romero, Santiago Royo-Sierra, Javier González-Galindo, Jinru Wei, Tania Sawaya, Astrid Van Wilder, Yu Qing Bai, Clas Rehnberg, Nils Janlöv, Reijo Sund, Walter P Wodchis, Irene Papanicolas, Enrique Bernal-Delgado

**Affiliations:** Data Science for Health Services and Policy Research Group, Institute for Health Sciences in Aragon (IACS), Aragón, Spain; Data Science for Health Services and Policy Research Group, Institute for Health Sciences in Aragon (IACS), Aragón, Spain; Data Science for Health Services and Policy Research Group, Institute for Health Sciences in Aragon (IACS), Aragón, Spain; Center for Health System Sustainability, Department of Health Services, Policy & Practice, Brown University School of Public Health, Providence, RI, United States; Center for Health System Sustainability, Department of Health Services, Policy & Practice, Brown University School of Public Health, Providence, RI, United States; Center for Health System Sustainability, Department of Health Services, Policy & Practice, Brown University School of Public Health, Providence, RI, United States; Institute of Health Policy, Management and Evaluation, University of Toronto, Toronto, Ontario, Canada; Institute for Better Health, Trillium Health Partners, Toronto, Ontario, Canada; Department of Learning, Informatics, Management and Ethics (LIME), Karolinska Institute, Stockholm, Sweden; Department of Learning, Informatics, Management and Ethics (LIME), Karolinska Institute, Stockholm, Sweden; Swedish Agency for Health and Care Services Analysis (Vårdanalys), Stockholm, Sweden; Institute of Clinical Medicine, School of Medicine, Faculty of Health Sciences, University of Eastern Finland, Kuopio, Finland; Welfare State Research Unit, Finnish Institute for Health and Welfare, Helsinki, Finland; Knowledge Management Unit, Kuopio University Hospital, Kuopio, Finland; Institute of Health Policy, Management and Evaluation, University of Toronto, Toronto, Ontario, Canada; Institute for Better Health, Trillium Health Partners, Toronto, Ontario, Canada; Center for Health System Sustainability, Department of Health Services, Policy & Practice, Brown University School of Public Health, Providence, RI, United States; Data Science for Health Services and Policy Research Group, Institute for Health Sciences in Aragon (IACS), Aragón, Spain

## Abstract

Outcomes after a hip repair in the older adult population are highly dependent on patients’ characteristics. However, contextual factors such as the hospital of treatment may have an impact not sufficiently studied. We aimed to elicit the effect of hospital providers on all-cause-adjusted mortality rates after hip fracture repair. Observational study on virtually all potentially eligible hip fracture patients treated in 2240 hospitals from Ontario (Canada), Aragon (Spain), Finland, Sweden, and the USA (40 states). The primary endpoint was the risk-adjusted all-cause mortality after hip repair measured 30 days and 180 days after surgery. Following a federated approach, GAMM-logit models were run for each region. Median odds ratio (MOR) were estimated to elicit the variation at hospital level. The study included 535 519 hip repairs. The overall predicted 30-day adjusted mortality rate was 40.5 per 1000 hip repair episodes; 136.3 per 1000 hip repair episodes in the 180-day adjusted mortality rate. 30- and 180-day adjusted mortality rates were larger within the regions than across regions. Variance in patients’ mortality at the hospital provider accounted for MOR: 1.43 in 30-day mortality and MOR: 1.35 in 180-day mortality. Beyond differences in the individual risk of death, our study found wide systemic variations in mortality rates in older adult patients exposed to hip fracture repair attributable to the hospital of treatment. Our results call for a reorientation of care pathways after hip repair in frail patients, both in the short- and the long-term.

## Introduction

Hip fractures among older adults are becoming a growing medical and social concern, resulting in increasing frailty, decreasing autonomy and quality of life, and higher risk of death—7% of those suffering from a hip fracture will die during the hospitalization stay and 30% will die within the year after the episode [1, 2].

The high death toll has been a matter of analysis in numerous studies. Patient characteristics such as advanced age, male gender, and the co-existence of other conditions have been shown to increase the risk of death [3]. Still, a substantial part of the variation in the risk of death has been attributed to medical practice, in particular, to delays in treatment, the type of surgical procedure, or prompt mobilization after hip repair [4–6]. Additionally, early discharge strategies have also been suggested to increase readmission and the risk of death [7]. Despite the wealth of literature, international studies comparing the effect of contextual factors, such as EUROHOPE [8], are scarce, particularly those analysing the care after a hip repair hospital episode. Studies of this kind could shed light on the importance of area-level factors in hip fracture outcomes, such as the availability of post-acute resources and services.

The International Collaborative on Costs, Outcomes and Needs in Care (ICCONIC) is interested in the comparative study of health systems performance among high-need frail patients. In a recent exploratory analysis on hip fracture repair outcomes, ICCONIC found large differences in mortality rates across countries; for example, in-hospital mortality rates ranged from 1.5% in Australia to 11.5% in England, and 30-day mortality ranged from 5.8% in Australia to 9.8% in England [9]. Likewise, the rate of readmissions at 30 days varied widely from 7.0% in England to 35.6% in Australia [10]. Based on aggregated data, these results raised the hypothesis that systemic differences across health systems may explain a substantial part of the variations in mortality rates after hip fracture repair besides individual and practice factors.

Building on the data from the ICCONIC network, we carried out a large-scale study to further examine this question. Using individual patient data, for each region, we sought to elicit the effect of hospital providers on all-cause-adjusted mortality rates at different intervals for patients presenting to the hospital with a hip fracture.

## Methods

This observational retrospective study linked individual data from patients aged 65 and over with an unplanned admission for primary hip fracture who underwent hip repair (i.e. pinning or replacement surgery) between 1 January 2017 and 31 December 2021. Patients with polytrauma and referred from other hospitals were excluded (Common Data Model (CDM) [11]). In addition, to obtain more precise estimates, those hospitals with less than 50 hip fracture episodes during the study period were excluded from the analyses (Fig. S1).

The study was based on virtually all potentially eligible hip fracture patients treated in five health systems: Ontario (CAN), Aragon (ESP), Finland (FIN), Sweden (SWE), and the USA-Medicare, this latter split out in 40 States, 44 geographical regions in total. The 44 regions acted as independent units for the modelling and representation of the results. Data were retrieved from administrative sources, population registries and electronic health records (EHR). In the case of Ontario (CAN), data were extracted from the Canadian Institute for Health Information Discharge Abstract Database (DAD), which captures administrative, clinical, and demographic information on hospital discharge; in Aragon (ESP), the data were extracted from BIGAN (*established by Executive Order SAN/1355/2018 as an element of the regional health information system*); in Finland, data were extracted from the PERFECT research database that is based on nationwide Finnish health registers such as Care Register for Health Care and Causes of Deaths Register [12]; in Sweden, data were extracted from the National Board of Health and Welfare; and in the USA, data were extracted from the Medicare database, managed by the Centers for Medicare & Medicaid Services (CMS). Table S1 provides additional detail on the different data sources in the study.

The primary endpoint in the study was the risk-adjusted all-cause mortality after hip repair measured 30 days and 180 days after surgery. In the analysis of the first time-period, we sought to elicit differences in adjusted mortality rates that could be associated with both the immediate post-surgery care and the short-term continuity of care after discharge. In the 180-day time-lapse, we sought to establish differences in adjusted mortality rates related to the long-term follow-up activity delivered to patients with hip repair after hip fracture. Our study uses all-cause mortality to capture the complex interaction between an older adult patient and the health system in a situation that boosts frailty.

Adjustment factors included patient attributes (i.e. 5-year age group, sex, 11 comorbidities relevant to the topic validated by an orthopaedic surgeon, and previous hospital admission), care process predictive variables (i.e. time to surgery, type of surgery, length of stay after surgery, need for an ICU stay, and month of treatment), contextual information (hospital provider defined statistically as a random effect for those non-observable or unknown effects triggered as a consequence of the index hospitalization). All the variables of interest were collected and defined in the CDM of the project [11]. Variables introduced in the final models were those that showed significant statistical association in most regions [i.e. those for which OR distribution across regions did not include 1 within their interquartile range (IQR)].

Following a federated approach [13], descriptive analyses and generalized additive multilevel logit models (GAMM) were implemented in Docker and Apptainer, deposited in a GitHub repository [14] and deployed in each participant node. After a data quality review, local results were compiled. A comparative analysis was conducted to identify those non-patient factors that had more influence on the 30- and 180-mortality rates. GAMM-logit models were run for each region (*n* = 44) to predict all-cause mortality rates 30 and 180 days after hip repair. All variables were modelled as binary or categorical except ‘length of stay after surgery’, modelled as continuous, and ‘time-to-surgery’, modelled as a smoothed non-parametric function. The ‘hospital provider effect’ was modelled as a random effect accounting for the latent clustering phenomenon for patients treated by the same hospital provider.

For each region, the median odds ratio (MOR) (and bootstrapped confidence interval) for 30-day and 180-day models was estimated to elicit the variation in mortality differences at the hospital provider level. MOR has to be interpreted as the median relative risk difference between the patients treated by the hospital provider with the highest risk and the patients assisted by the hospital provider with the lowest risk; so, for example, a MOR value of 1.5 would inform on a patient’s risk 1.5 times higher in the hospital provider with the highest risk as compared with the one with the lowest risk, irrespective of patients differences [15, 16].

The analysis was programmed using R programming language [17], using the packages ‘*mgvc*’ for GAMM, ‘*broom*’ and ‘*performance*’ in assessing the statistical models and ‘*Quarto*’ in building the local reports produced for each region [18–21]. The mathematical notation of the statistical model can be found in the Mathematical Notation section of the Supplementary Material. Figure S2 shows a box and whiskers plot (calculated by Tukey's method [22]) with the distribution of the OR for the 44 models weighted by the hip repair volume in each region. The figure informs what predictors were more likely to explain the differences in hip fracture mortality in all 44 regions. The goodness of fit was assessed using the Akaike Information Criteria (AIC) and the distribution of mean residuals [23] (Fig. S3).

## Results

The study included 590 938 hospital hip fracture episodes in patients aged 65 and older, which contained 535 519 hip repairs delivered in 2240 hospital providers from 44 regions based in five health systems. The number of all-cause deaths at 30 days was 33 617 and at 180 days was 92 551 after hip repair.

The overall 30-day all-cause mortality rate was 62.8 per 1000 hip repair episodes, and the 180-day all-cause mortality rate was 172.8 per 1000 hip repair episodes. The overall predicted median 30-day adjusted mortality rate was 40.5 per 1000 hip repair episodes (IQR: 34.8–47.7) and 136.3 per 1000 hip repair episodes (IQR: 119.5–152.0) in the 180-day adjusted mortality rate (Table 1).

**Table 1.** Sample, years 2017–2021

Health system	N regions	N hospitals [range]	N hospitalization episodes [range]	N hip repair episodes [range]	N deaths in 30 days after hip repair [range]	N deaths in 180 days after hip repair [range]
CAN	1	77	54 391	48 974	3291	8070
ESP	1	11	8207	7453	538	1201
FIN	1	27	24 338	22 653	1710	3968
SWE ^a^	1	52	14 080	13 039	1012	2406
USA ^b^	40	2073 [5–196]	489 922 [872–47 747]	443 400 [777–42 941]	27 066 [22–2623]	76 906 [64–7423]
Total	44	2240	590 938	535 519	33 617	92 551

a Sweden figures refer to 2016–2017.

b USA data: between brackets the range of values across the 40 regions analysed.

Patients’ variables consistently and significantly associated with the mortality rates in the majority of regions were: in the case of 30-day mortality, being a man (OR_P50_ 1.82 [1.47–2.38]), older than 89 years (OR_P50_ 4.34 [2.65–8.45]), with a previous admission (OR_P50_ 1.28 [1.02–1.66]), congestive heart failure (OR_P50_ 1.6 [1.16–1.94]), kidney disease (OR_P50_ 1.84 [1.15–2.45]), peripheral vascular disease (OR_P50_ 1.38 [1.10–1.64]), and dementia (OR_P50_ 2.26 [1.86–2.61]) were found risk factors in at least 41 regions.

In the case of 180-day mortality, being a man (OR_P50_ 1.69 [1.52–1.89]), older than 74 years, the higher the older (OR_P50_ 1.48; [1.05–1.93] in patients between 75 and 79; and OR_P50_ 4.04; [2.55–5.21] in patients aged 90 and older), with a previous admission (OR_P50_ 1.57; [1.36–1.77]), congestive heart failure (OR_P50_ 1.56; [1.30–1.93]), kidney disease (OR_P50_ 2.04; [1.68–2.54]) and dementia (OR_P50_ 2.6; [1.99–3.06]), and having received pinning surgery (OR_P50_ 1.67; [1.03–2.22]) were found associated in at least 41 regions (Table S2).

Overall differences in the median predicted mortality ranged between 30.0 and 51.9 per 1000 hip repair episodes in the 30-day adjusted mortality rate, and between 107.8 and 157.4 in the 180-day adjusted mortality rate. By health system, the predicted median for the 30-day adjusted mortality rate was 40.5 per 1000 hip repair episodes in Canada, 49.3 per 1000 hip repair episodes in Spain, 38.8 per 1000 hip repair episodes in Finland, 51.9 per 1000 hip repair episodes in Sweden, and 40.2 [30.0–49.6] per 1000 hip repair episodes in the USA. In the latter case, the predicted median for a 180-day adjusted rate was 132.4 per 1000 hip repair episodes in Canada, 128.5 per 1000 hip repair episodes in Spain, 142.9 per 1000 hip repair episodes in Finland, 155.4 per 1000 hip repair episodes in Sweden, and 135.7 [107.8–157.4] per 1000 hip repair episodes in the USA (Table S3).

**Figure 1.**
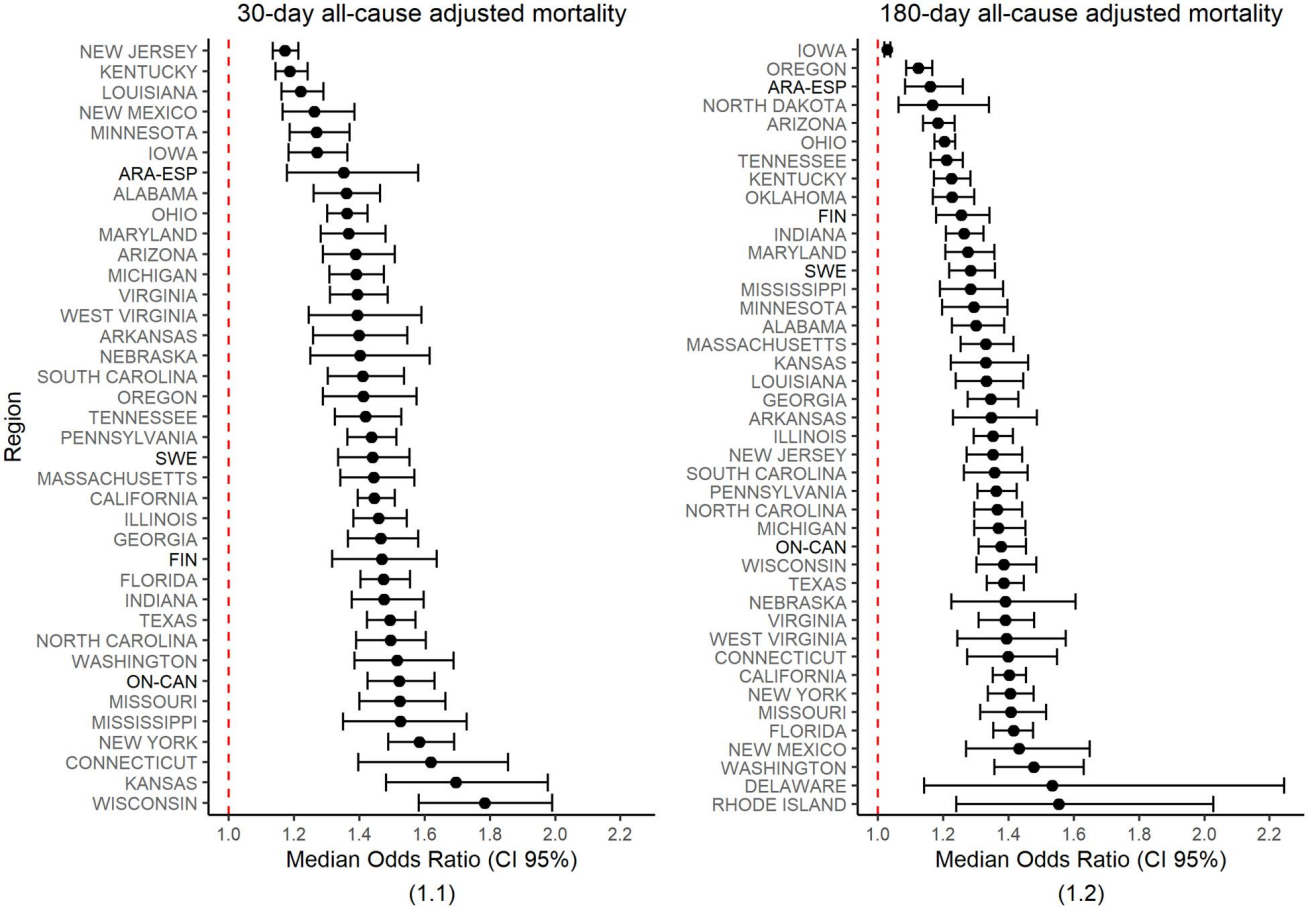
Differences in hospitals median odds ratio across regions. (1.1) 30-day all-cause adjusted mortality. (1.2) 180-day all-cause adjusted mortality. Median odds ratio (MOR, bootstrapped confidence interval) by region. The MOR for each region is represented as a point range with error bars showing the bootstrapped confidence interval. Regions are sorted by MOR value from highest to lowest. Non-USA regions’ labels are bolded to facilitate differentiation. The red dashed vertical line is at value 1, which is the minimum possible MOR value. Extreme outliers are excluded—(1.1): Alaska, Delaware, Nevada, North Dakota, Oklahoma and Rhode Island (82 hospitals in total); and (1.2) Alaska and Nevada (24 hospitals in total).

When it comes to the variation in patients’ mortality at the hospital provider level, the overall MOR for the 30-day all-cause mortality model accounted for 1.43, while the overall MOR for the 180-day all-cause mortality model accounted for 1.35. The MOR for the 30-day all-cause adjusted mortality ranged from 1.17 in New Jersey to 1.78 in Wisconsin; in turn, the MOR for 180-day all-cause adjusted mortality ranged from 1.03 in Iowa to 1.55 in Rhode Island (Fig. 1 and Table S4).

**Figure 2.**
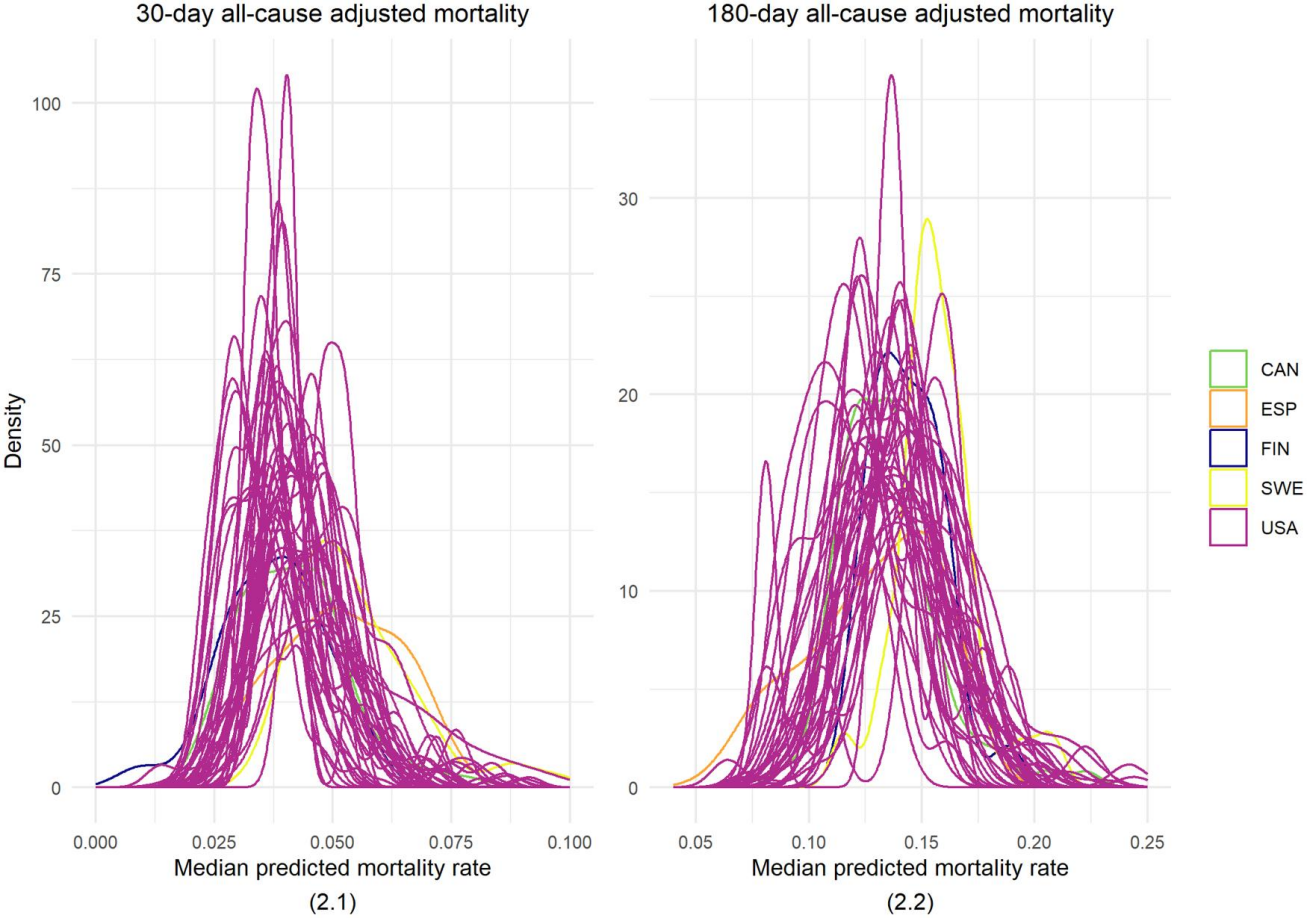
Predicted median for all-cause mortality rate after hip repair: within and across regions variation. (2.1) 30-day all-cause adjusted mortality. (2.2) 180-day all-cause adjusted mortality. Probability density function plot of the median predicted mortality rate per hospital in each region. Each density curve represents the distribution of the median values for all hospitals within a region and is coloured by the health system. Wide and overlapping curves show larger differences within regions than between regions. Extreme outliers are excluded—(2.1) Ten hospitals (seven below and three above, i.e. 0.45% of the total number of hospitals) which include 934 (0.17% of total surgeries) hip repair episodes. (2.2) Ten hospitals (eight below and two above, i.e. 0.45% of the total number of hospitals), which include 938 (0.18% of total surgeries) hip repair episodes.

When it comes to within vs. across regional differences, both 30- and 180-day adjusted mortality rates were larger within regions than between regions. For the 30-day adjusted mortality rate, the regions' highest to lowest predicted median rate ratio was 1.73, while the within-region extreme ratio was 2.47. For the 180-day adjusted mortality rate, the highest to lowest predicted median rate ratio was 1.46, while the within-region extreme ratio was 2.06 (Fig. 2 and Table S3).

**Figure 3.**
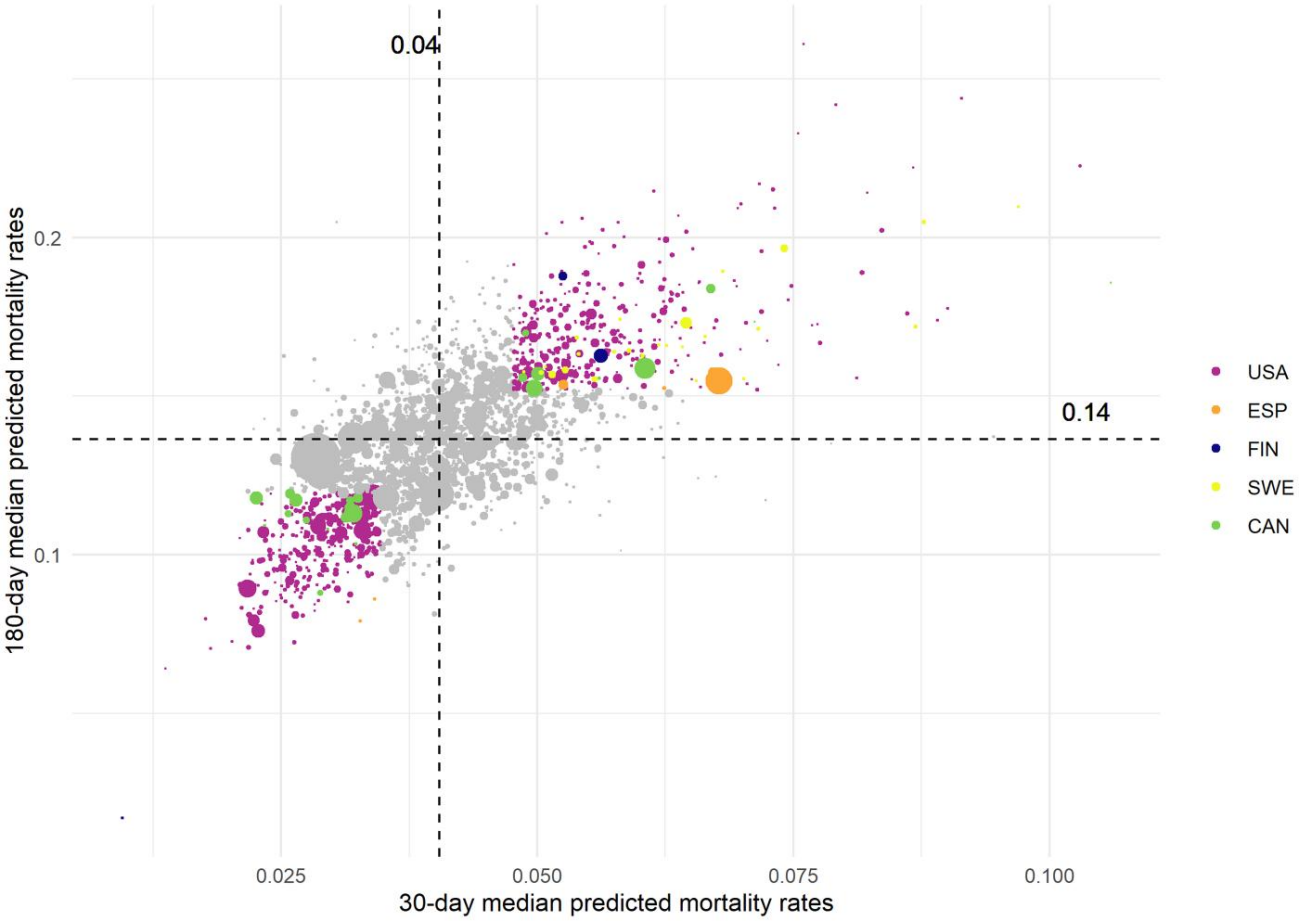
30-day predicted mortality rates vs. 180-day predicted mortality rates. 30-day predicted mortality rates versus 180-day predicted mortality rates by hospital. Each dot represents a hospital and is coloured by the health system. Coloured dots flag those hospital providers outside the IQR of the median predicted value in both indicators. The size of the dot represents the number of surgeries performed in each hospital. The dashed vertical line marks the median predicted 30-day mortality rate, which is 0.04 per hip repair episode, and the dashed horizontal line marks the median predicted 180-day mortality rate, which is 0.14 per hip repair episode. Extreme outliers are excluded: seven hospitals in Alaska and one hospital in Canada.

In Fig. 3, coloured dots flag those hospital providers outside the IQR of the median predicted value in both indicators. 365 hospitals out of 2232 were flagged as having larger predicted mortality at both 30- and 180-day, and 371 hospitals out of 2232 had less adjusted mortality in both indicators.

## Discussion

This observational study included 590 938 hospital hip fracture episodes in patients aged 65 and older 535 519 hip repairs delivered in 2240 hospital providers based in 44 regions from five high-income health systems.

The variation of adjusted mortality across hospital providers was found to be high, with a 43% risk increase in the patients treated in the hospital provider with the highest 30-day adjusted mortality and a 35% risk increase in the hospital provider with the highest 180-day adjusted mortality. In addition, our study has shown differences in predicted mortality among hospital providers within a region, larger than the differences across regions [24].

The large MOR in the 30-day all-cause mortality model suggests that, beyond patient characteristics, mainly being a man aged 90 and older, with previous admissions and suffering from congestive heart failure, kidney disease, and dementia, there are latent factors at the hospital level that may have been triggered during the hospital stay (e.g. quality and safety events) or in the immediate period after discharge (e.g. care discontinuity, access barriers to home care). In addition, the large MOR in the 180-day all-cause mortality model suggests the influence of other latent factors, in addition to those in the index episode, that may be related to, for example, the care pathways to which the patient is exposed and then, care fragmentation or the lack of coordination between health care and social care sectors.

### Coherence with existing evidence

There is little cross-national evidence to compare our MOR estimates. Nonetheless, a local study in Denmark found a median odds ratio (MOR) of 1.18 (95% CI: 1.12–1.25) in 32 hospitals, a bit lower effect than the one observed in our study, although the variance partitioning coefficient (VPC), another estimand for the cluster effect, was similar to ours. Another study on 44 Swedish hospitals found a similar VPC [25, 26].

When it comes to individual factors, a recent meta-analysis on early hip repair mortality identified men aged 85 and older with multiple comorbidities admitted from another setting as those patients at higher risk of 30-day mortality [27]. Evidence from a scoping review showed that the month of treatment, the time-lapse between diagnosis and surgery, the length of stay, the type of anaesthesia or the volume of cases (hospital, surgeon, and nursing volume) were associated with post-discharge mortality [28]. Other local studies showed the relevance of organizational factors in hip repair outcomes, particularly those affecting early treatment decisions, such as the surgical volume and the local concentration of treatments [29, 30]. Our large-scale study has nuanced the relevance of some of those factors; thus, an association between time from admission to treatment and mortality was found, although it was observed in just 10 regions in 30-day mortality and 16 in 180-day mortality; once the sample of hospital providers was divided into quintiles of the volume of surgeries, the median predicted rate of mortality was similar across quintiles (Table S5), finding that is coherent with previous research [31].

When it comes to the policy implications of this work, as Fig. 1 exhibits, there are some regions where the MOR value in 30-day mortality is notably smaller than the MOR value in 180-day mortality (e.g. New Mexico) and the other way around (e.g. Finland). The correct interpretation of the relative importance of one or another could have a direct influence on policy action, leading to prioritizing remedies to control specific factors. Thus, a higher value in 30-day mortality would underscore factors such as inadequate surgical indications, surgical quality of care [32, 33], early discharge policies, and discontinuity of care after discharge [34]; on the contrary, a higher MOR value in 180-day mortality would point out to systematic differences in the long-run follow-up of the patient, the effective access to adequate resources (e.g. rehabilitation wards), and coordination with social care [35]. As a matter of example, Finland's healthcare system has improved immediate care [36]. However, Finnish hospitals exhibit relatively high mortality and readmission rates within the first-year post-fracture emphasizing the critical nature of long-term care integration as well as the need for postoperative rehabilitation services [37].

On a different note, from the point of view of performance monitoring, Fig. 3 informs us on the possibility of plotting all the hospitals using four benchmark quadrants. Thus, those hospitals in the first quadrant would show higher predicted mortality rates in both 30- and 180-day mortality rates, flagging potential performance failures in both short-term and long-term care, while hospitals placed in the third quadrant could well be benchmarks for the rest of their peers. Thus, the present study on the variation in 30- and 180-day adjusted mortality rates of 2240 hospital providers across 44 different regions could well offer an opportunity to establish an international benchmark on hip repair health outcomes at three meaningful levels of assessment—hospital, region, and health system.

### Limitations

#### Data issues

This study builds on observational administrative, registry, and EHR data. Although original data were extracted from well-known validated data sources and dataset quality was assessed before running the models, some data flaws may still affect the estimands. Although the main variables of our study (e.g. age groups, sex, death, hospital provider, type of surgery or time from admission to surgery) were found homogeneously recorded across health systems, comorbidities may suffer from differential recording. To analyse any potential impact, we studied the difference in the number of comorbidities per hospital episode and health system. The median number of those comorbidities included in the models ranged from a median of 1.2 comorbidities in the Swedish hospitals to 2.4 comorbidities in the Aragonese hospitals (Spain); in turn, the median was 1.3 comorbidities in Ontario (Canada), 2.0 comorbidities in Finland, and 2.3 comorbidities in the USA (Fig. S4). Despite these differences, no association (r^2^=0.01) was found between the number of comorbidities recorded and 30-day and 180-day adjusted mortality rates (Fig. S5).

In addition, some potential predictors of mortality have not been included in the models as they were not registered in all the participant health systems. This was the case for socioeconomic level, race, type of anaesthesia, and antiplatelet or anticoagulant treatment before the episode of interest. In the case of socioeconomic status and race, only some health systems collect this data on a regular basis. Whether both variables would have impacted the risk and MOR estimates is contentious. A recent meta-analysis showed that although minority groups faced delays in access to treatment, experienced more readmissions, longer lengths of stay, and suffered from more perioperative complications, 30-day mortality appeared not to be higher than in white populations [38]. In the case of the type of anaesthesia and the case of antiplatelet or anticoagulant treatment, we may argue that their potential effect is already captured with the use of other predictors in the models; in the case of anaesthesia, the effect may be mediated by the type of surgery, while the use of anticoagulant treatment is mediated by a longer time from admission to surgery.

Finally, although Swedish estimates may be biased given that the data come from 2 years instead of 5, as in the other health systems, 30-day mortality rates reported from a nationwide cohort study in Sweden from 1998 to 2017 were found to be consistent with observed rates in the present study [39].

#### Size heterogeneity across units of analysis

Our sample includes 2240 hospitals whose volume ranges from 50 to 3961 hip fracture episodes, thus with great potential for extra heterogeneity overestimating MOR. Hospitals that did not deliver at least 50 hip fracture episodes were excluded from the analyses to reduce this potential effect. The exclusion affected 1328 unique hospitalizations (2%) and 93 hospitals (55%) in Canada, 361 hospitalizations (4%) and 8 hospitals (42%) in Spain, 177 hospitalizations (1%) and 23 hospitals (31%) in Sweden, 12509 hospitalizations (3%) and 734 hospitals (26%) in the USA, and none in Finland (Fig. S1). Besides this strategy, we tested whether our estimates suffered from extra-heterogeneity by calculating the ratio between the restricted maximum likelihoods of the fixed effects and the random effects regression models in each local participant's analysis. No ratio values were above 2, and consequently, although the observed extra-heterogeneity, it is unlikely for MOR values to be overestimated [40]. While excluding hospitals with fewer than 50 operations enhanced the statistical stability of our analysis, this criterion resulted in the exclusion of smaller facilities, limiting the generalizability of our findings to those larger, higher-volume hospitals.

#### Unmeasured confounding

Our cross-sectional approach does not allow discerning whether changes in clinical practice or the implementation of specific policies along the study may have been influencing our estimates. However, we do expect that any potential change in either practice or policies, if differential across hospitals and if predictive of mortality, have been captured by the random part of our models making MOR values unbiased.

Although most of the variance in hospital mortality rates is at individual level, potential selection bias in smaller hospitals and residual confounding at sub-regional, regional, and national level is still possible. Any use of our estimates to inform local policies or to conduct international hospital-level benchmarking studies should be interpreted considering contextual factors not measured in this study.

## Conclusions

Beyond differences in the individual risk of death, our study found wide systemic variations in mortality rates in older adult patients exposed to hip fracture repair attributable to the hospital of treatment. The variance in mortality that is at the hospital level was significantly larger in the immediate post-acute care, an indication that immediate transmural care is most likely the biggest contributor to between-hospital variation in outcomes. Our results call for a reorientation of care pathways after hip repair in frail patients, both in the short- and the long-term.

## Supplementary Material

ckaf074_Supplementary_Data

## Data Availability

The data will be shared on reasonable request to the corresponding author subject to third-party constraints. The study includes 590 938 hospital hip fracture episodes in patients aged 65 and older, with 535 519 hip repairs delivered in 2240 hospitals from 44 regions based in five health systems. Differences in death risk were larger within regions than across regions, likely stressing the importance of local contextual factors when explaining differences in hip fracture repair mortality. Beyond differences in the individual risk of death, our study found wide systemic variations in mortality rates that can be attributed to contextual or systemic factors. We found that depending on the hospital of treatment 30-day mortality exhibited a 43% risk increase; a 35% risk increase in 180-day mortality. The large contribution of systemic factors elicited by the MOR values, call for a re-orientation of the services delivered to this kind of patients from a hospital-centric approach to an approach based on the whole care pathway.
