# Supplementary material for: Examining systemic differences in mortality after hip repair: a comparative analysis of 30- and 180-day adjusted mortality rates in five health systems
Source: Eur J Public Health. 2025 Jun 26;35(5):855–61. doi: 10.1093/eurpub/ckaf074 (PMC12529295; doi:10.1093/eurpub/ckaf074)
Supplement: ckaf074_Supplementary_Data [file ckaf074_supplementary_data.zip › ckaf074_Supplementary_Data/ejph-2025-02-om-0094-File005.docx]

# Supplementary material

**Table S1. Data sources**

| **Health system** | **Population 65 and older potentially covered by the system** | **Population 65 and older with hip fracture (expected prevalence)** | **Data source -**  **(official name, curator, short description of the content, any local study assessing quality)** | **Population 65 and older with hip fracture covered in this data source** |
| --- | --- | --- | --- | --- |
| Ontario (Canada) | As of January 1, 2017, 2,404,307 Ontario residents aged 65 to 105 years were covered by the Ontario Health Insurance Plan (OHIP), representing 16.5% of the Ontario population. | An age-standardized hip fracture rate of 21.0 per 10,000 adults **aged 50 years** and older was reported for the fiscal year 2019/20 (<https://osteostrategy.on.ca/wp-content/uploads/OOS-Provincial-Performance-Data-Technical-Report-Mar-17-23.pdf>)**.** | Canadian Institute for Health Information Hospital Discharge Abstract Database (DAD). DAD captures administrative, clinical and demographic information on hospital discharges  (<https://www.cihi.ca/en/discharge-abstract-database-dad-metadata>). | A total of 53,768 hip fracture-related hospital admissions were identified from calendar years 2017 to 2021 in Ontario, Canada. |
| Aragon (Spain) | 289,603 inhabitants 65 years old or older (21.83% of the population). | 664 fractures  /100,000/inhabitants/year (<https://revistasanitariadeinvestigacion.com/estudio-epidemiologico-de-las-fracturas-de-cadera-en-la-poblacion-mayor/?utm_content=cmp-true>). | BIGAN, Institute for Health Sciences in Aragón (<https://bigan.iacs.es/en/home>). BIGAN is a technological platform that integrates all Aragón (Spain) health system data for healthcare managers, educators, and researchers. BIGAN’s data catalogue can be accessed at <https://bigan.iacs.es/en/services/data-catalogue> | 8,568 hospital admissions due to hip fractures from 2017 to 2021 (515 fractures/100,000/inhabitants/year). |
| Finland | Total population of 65+ years old in mainland Finland, 1.14million (20.9%) in the beginning of 2017, 1.27million (23.1%) in the end of 2021. | Age-standardized rate in 2018 (50+ years old) for men 20 per 10,000 and for women 25 per 10,000 (<https://doi.org/10.1007/s00198-021-05906-6>) . | PERFECT hip fracture database linking several nationwide administrative registers, hip fractures identified from the Care Register for Health Care (<https://doi.org/10.3109/07853890.2011.586360>) (<http://doi.org/10.1016/j.socscimed.2020.113611>). | All 65+ years old with hip fracture included, totally 24,338 new hip fractures between 2017-2021. |
| Sweden | Universal health coverage for all citizens applies, so the entire population 65+ included in the system | 789 fractures per 100,000 inhabitants 65+ in 2016 | The Patient Register by the National Board of Health and Welfare provides the basis for statistics on diseases and treatments in Swedish specialised care and include all completed inpatient stays since 1964 (nationwide since 1987) (<https://www.socialstyrelsen.se/en/statistics-and-data/registers/national-patient-register/>).  The National Cause of Death Register by the National Board of Health and Welfare provides the basis for official cause-of-death statistics in Sweden (<https://www.socialstyrelsen.se/en/statistics-and-data/registers/national-cause-of-death--register/>). | 14,257 hospital admissions with procedures due to hip fractures from 2016-2017. |
| Medicare Fee-for-Service  40 States (USA) | In January 2017, 30.15 million beneficiaries accounted for 9.81% of the total population (range: 0.08–3.04 million; 6.43%–15.19%).  In December 2021, 29.28 million beneficiaries represented 9.36% of the total population (range: 0.09–3.14 million; 7.00%–15.20%). | In 2019, the rate of hip fracture hospitalizations among adults aged 65 and older was 564.5 per 100,000, with a higher rate observed in women (668.9 per 100,000) compared to men (415.7 per 100,000) (<https://doi.org/10.1177/08982643221132450>). | Medicare Fee-for-Service (FFS) Data from the Centers for Medicare & Medicaid Services (CMS) (<https://www.cms.gov/>). The FFS data, accessible through the Chronic Conditions Data Warehouse (CCW), provides comprehensive claims and enrollment data on healthcare utilization and outcomes (<https://www2.ccwdata.org/web/guest/home>). | A total of 502,431 new hip fractures were identified across 40 states in the USA between 2017 and 2021, with state-level hospital admissions ranging from 876 to 47,982. |

**Table S2. GAMM models: Distribution of estimands**

**Table S2.1. Risk of death at 30 days**

| **Variable** | **Percentile 50** | **Percentile 25** | **Percentile 75** | **Lower whisker** | **Upper whisker** |
| --- | --- | --- | --- | --- | --- |
| **Patient attributes** | | | | | |
| Age group | | | | | |
| 14: 65-69 yo^a^ | Reference | | | | |
| 15: 70-74 yo | 1.32 | 1.19 | 1.78 | 0.53 | 2.61 |
| 16: 75-79 yo | 1.63 | 1.38 | 2.19 | 0.43 | 3.15 |
| 17: 80-84 yo | 2.1 | 1.82 | 2.86 | 0.97 | 4.16 |
| 18: 85-89 yo | 2.76 | 2.44 | 3.66 | 0.84 | 5.23 |
| 19: >= 90 yo | 4.34 | 4.05 | 5.81 | 2.65 | 8.45 |
| Sex | | | | | |
| Male | Reference | | | | |
| Female | 0.55 | 0.51 | 0.6 | 0.42 | 0.68 |
| Comorbidities | | | | | |
| Cerebrovascular disease | 2.73 | 2.09 | 3.56 | 0 | 5.37 |
| Chronic kidney disease | 1.84 | 1.61 | 1.97 | 1.15 | 2.45 |
| Congestive heart failure | 1.6 | 1.46 | 1.69 | 1.16 | 1.94 |
| Coronary artery disease | 1.42 | 1.29 | 1.62 | 0.85 | 2.09 |
| Dementia | 2.26 | 2.13 | 2.36 | 1.86 | 2.61 |
| Liver disease | 1.8 | 1.33 | 2.54 | 0 | 4.02 |
| Obesity overweight | 0.74 | 0.72 | 0.89 | 0.52 | 1.11 |
| Parkinson Huntington diseases | 1.15 | 1.02 | 1.35 | 0.78 | 1.73 |
| Peripheral vascular disease | 1.38 | 1.27 | 1.43 | 1.1 | 1.64 |
| Tobacco/COPD | 1.09 | 1.06 | 1.21 | 0.85 | 1.33 |
| Traumatic brain injury | 1.47 | 1.16 | 1.81 | 0.63 | 2.7 |
| Background | | | | | |
| Previous hospital admission | 1.28 | 1.22 | 1.41 | 1.02 | 1.66 |
| **Care process** | | | | | |
| ICU admission | 2.27 | 1.98 | 2.72 | 1 | 3.2 |
| Time from admission to surgery ^b^ | 0.27 | 0.07 | 0.53 | 0 | 0.87 |
| Length of stay after surgery | 1.02 | 1 | 1.03 | 0.95 | 1.06 |
| Type of surgery | | | | | |
| Total replacement | Reference | | | | |
| Partial replacement | 1.58 | 1.23 | 1.89 | 0.76 | 2.75 |
| Hip pinning | 1.7 | 1.23 | 1.98 | 0.88 | 3.02 |
| Other | 1.77 | 1.29 | 2.3 | 0 | 3.4 |
| Month of treatment | | | | | |
| 1: January | Reference | | | | |
| 2: February | 0.9 | 0.78 | 1.05 | 0.47 | 1.24 |
| 3: March | 0.97 | 0.82 | 1.08 | 0.5 | 1.39 |
| 4: April | 0.92 | 0.81 | 1 | 0.56 | 1.26 |
| 5: May | 0.85 | 0.77 | 0.97 | 0.51 | 1.25 |
| 6: June | 0.9 | 0.88 | 1.02 | 0.7 | 1.23 |
| 7: July | 0.97 | 0.9 | 1.14 | 0.71 | 1.46 |
| 8: August | 0.94 | 0.84 | 1.1 | 0.51 | 1.49 |
| 9: September | 1.02 | 0.92 | 1.13 | 0.7 | 1.42 |
| 10: October | 1.02 | 0.93 | 1.2 | 0.67 | 1.52 |
| 11: November | 1.16 | 0.94 | 1.27 | 0.47 | 1.63 |
| 12: December | 1.08 | 0.98 | 1.22 | 0.64 | 1.59 |

Note: ^a^ yo=years old.

^b^ All the values represent Odds Ratios except in time from admission to surgery, where the p-values distribution is exhibited.

Interpretation note: variables with an upper whisker below one are considered protective factors, while variables with a lower whisker above one are considered risk factors.

**Table S2.2. Risk of death at 180 days**

| **Variable** | **Percentile50** | **Percentile25** | **Percentile75** | **Lower whisker** | **Upper whisker** |
| --- | --- | --- | --- | --- | --- |
| **Patient attributes** | | | | | |
| Age group | | | | | |
| 14: 65-69 yo^a^ | Reference | | | | |
| 15: 70-74 yo | 1.21 | 1.09 | 1.29 | 0.83 | 1.58 |
| 16: 75-79 yo | 1.48 | 1.36 | 1.6 | 1.05 | 1.93 |
| 17: 80-84 yo | 1.86 | 1.72 | 1.94 | 1.47 | 2.17 |
| 18: 85-89 yo | 2.39 | 2.18 | 2.62 | 1.71 | 3.2 |
| 19: >= 90 yo | 4.04 | 3.66 | 4.56 | 2.55 | 5.21 |
| Sex | | | | | |
| Male | Reference | | | | |
| Female | 0.59 | 0.57 | 0.62 | 0.53 | 0.66 |
| Comorbidities | | | | | |
| Cerebrovascular disease | 1.96 | 1.54 | 2.29 | 0.96 | 3.38 |
| Chronic kidney disease | 2.04 | 1.93 | 2.23 | 1.68 | 2.54 |
| Congestive heart failure | 1.56 | 1.47 | 1.63 | 1.3 | 1.83 |
| Coronary artery disease | 1.26 | 1.15 | 1.32 | 0.98 | 1.43 |
| Dementia | 2.6 | 2.4 | 2.7 | 1.99 | 3.06 |
| Liver disease | 1.78 | 1.42 | 2.35 | 0.41 | 3.41 |
| Obesity overweight | 0.84 | 0.8 | 0.88 | 0.68 | 0.95 |
| Parkinson Huntington diseases | 1.25 | 1.13 | 1.34 | 0.91 | 1.62 |
| Peripheral vascular disease | 1.25 | 1.15 | 1.29 | 0.98 | 1.45 |
| Tobacco/COPD | 1.16 | 1.11 | 1.28 | 0.96 | 1.45 |
| Traumatic brain injury | 1.37 | 1.21 | 1.55 | 0.86 | 1.97 |
| Background | | | | | |
| Previous hospital admission | 1.57 | 1.5 | 1.62 | 1.36 | 1.77 |
| **Care process** | | | | | |
| ICU admission | 1.54 | 1.4 | 1.79 | 1 | 2.14 |
| Time from admission to  surgery ^b^ | 0.14 | 0.01 | 0.28 | 0 | 0.65 |
| Length of stay after surgery | 1.03 | 1.01 | 1.04 | 0.97 | 1.07 |
| Type of surgery | | | | | |
| Total replacement | Reference | | | | |
| Partial replacement | 1.7 | 1.35 | 1.77 | 1 | 2.2 |
| Hip pinning | 1.67 | 1.41 | 1.8 | 1.03 | 2.22 |
| Other | 1.71 | 1.32 | 1.96 | 0.64 | 2.38 |
| Month of treatment | | | | | |
| 1: January | Reference | | | | |
| 2: February | 0.92 | 0.88 | 1.02 | 0.68 | 1.21 |
| 3: March | 0.95 | 0.91 | 1.03 | 0.81 | 1.18 |
| 4: April | 0.91 | 0.87 | 0.99 | 0.78 | 1.16 |
| 5: May | 0.93 | 0.86 | 0.99 | 0.73 | 1.17 |
| 6: June | 1.11 | 0.97 | 1.23 | 0.69 | 1.56 |
| 7: July | 1.1 | 1.03 | 1.22 | 0.79 | 1.47 |
| 8: August | 1.16 | 1.05 | 1.24 | 0.89 | 1.49 |
| 9: September | 1.15 | 1.03 | 1.21 | 0.85 | 1.48 |
| 10: October | 1.14 | 1.02 | 1.19 | 0.8 | 1.43 |
| 11: November | 1.1 | 0.99 | 1.18 | 0.79 | 1.4 |
| 12: December | 1.08 | 0.98 | 1.14 | 0.82 | 1.36 |

Note: ^a^ yo=years old.

^b^ All the values represent Odds Ratios except in time from admission to surgery, where the p-values distribution is exhibited.

Interpretation note: variables with an upper whisker below one are considered protective factors, while variables with a lower whisker above one are considered risk factors.

**Table S3.** **Predicted median 30-day and 180-day all-cause adjusted mortality rate (per 1,000 hip repair episodes)**

| **Region** | **Health System** | **30-day adjusted mortality rate [range]** | **180-day adjusted mortality rate [range]** |
| --- | --- | --- | --- |
| ON-CAN | CAN | 40.5 [22.3-78.7] | 132.4 [88.0-222.7] |
| ARA-ESP | ESP | 49.3 [32.7-67.8] | 128.5 [79.1-157.7] |
| FIN | FIN | 38.8 [9.5-56.2] | 142.9 [120.0-187.9] |
| SWE | SWE | 51.9 [36.9-97.0] | 155.4 [113.8-209.8] |
| ALABAMA | USA | 47.7 [21.0-61.9] | 150.9 [105.3-185.3] |
| ARIZONA | USA | 35.5 [22.4-48.0] | 107.8 [83.0-142.1] |
| ARKANSAS | USA | 49.5 [39.4-71.9] | 147.6 [112-222.5] |
| CALIFORNIA | USA | 31.2 [17.7-60.2] | 112.8 [70.5-168.8] |
| CONNECTICUT | USA | 34.5 [21.0-52.1] | 122.5 [90.5-165.1] |
| DELAWARE | USA | 36.3 [26.4-45.5] | 136.6 [80.9-161.7] |
| FLORIDA | USA | 41.0 [20.2-75.5] | 137.0 [72.4-232.8] |
| GEORGIA | USA | 39.5 [24.1-62.4] | 145.9 [114.0-197.2] |
| ILLINOIS | USA | 41.2 [21.8-90.0] | 135.0 [70.8-195.7] |
| INDIANA | USA | 46.5 [26.0-86.1] | 145.2 [114.8-186.1] |
| IOWA | USA | 44.7 [33.6-63.7] | 129.7 [112.0-160.8] |
| KANSAS | USA | 39.7 [26.4-62.5] | 139.5 [94.4-178.1] |
| KENTUCKY | USA | 40.1 [32.9-79.1] | 140.0 [115.1-241.9] |
| LOUISIANA | USA | 38.4 [25.0-69.9] | 134.5 [90.2-210.7] |
| MARYLAND | USA | 35.5 [13.7-48.7] | 131.5 [64.1-163.8] |
| MASSACHUSETTS | USA | 39.4 [29.4-59.7] | 123.5 [85.1-161.9] |
| MICHIGAN | USA | 49.6 [30.7-86.7] | 147.4 [97.2-222.1] |
| MINNESOTA | USA | 41.5 [26.6-57.8] | 139.8 [96.5-178.9] |
| MISSISSIPPI | USA | 36.4 [23.6-57.1] | 134.2 [88.5-171.9] |
| MISSOURI | USA | 47.2 [29.4-76.8] | 153.1 [102.1-204.8] |
| NEBRASKA | USA | 35.8 [27.6-76.0] | 126.6 [90.2-177.8] |
| NEVADA | USA | 35.3 [30.5-44.0] | 124.2 [105.8-154.5] |
| NEW JERSEY | USA | 40.3 [31.2-55.3] | 136.9 [93.4-187.2] |
| NEW MEXICO | USA | 30.0 [22.4-70.5] | 139.4 [101.3-192.3] |
| NEW YORK | USA | 35.2 [21.7-70.3] | 121.9 [76.1-173.1] |
| NORTH CAROLINA | USA | 41.8 [22.9-62.3] | 142.8 [98.2-179.9] |
| NORTH DAKOTA | USA | 39.1 [33.9-50.9] | 119.3 [91.3-140.7] |
| OHIO | USA | 46.7 [22.9-91.4] | 154.4 [76.7-243.9] |
| OKLAHOMA | USA | 45.8 [30.3-83.6] | 150.7 [95.3-202.3] |
| OREGON | USA | 31.5 [24.0-50.7] | 116.7 [94.5-161.0] |
| PENNSYLVANIA | USA | 42.2 [27.2-81.7] | 139.9 [80.8-206.9] |
| RHODE ISLAND | USA | 40.0 [28.0-54.5] | 157.4 [81.3-188.5] |
| SOUTH CAROLINA | USA | 42.1 [23.2-62.3] | 140.6 [89.4-200.3] |
| TENNESSEE | USA | 41.0 [26.6-69.5] | 140.6 [99.8-202.4] |
| TEXAS | USA | 38.5 [21.8-65.2] | 137.9 [81.1-196.4] |
| VIRGINIA | USA | 43.6 [31.7-65.8] | 139.6 [87.5-186.3] |
| WASHINGTON | USA | 41.6 [28.3-64.3] | 122.6 [82.3-177.3] |
| WEST VIRGINIA | USA | 46.7 [32.7-77.4] | 142.7 [100.4-172.7] |
| WISCONSIN | USA | 49.0 [24.9-94.5] | 133.6 [87.4-214.1] |

Note: Extreme outliers are excluded: Alaska

**Table S4. Median Odds Ratio (MOR) and intraclass correlation coefficient (ICC) amongst regions**

**Table S4.1. 30-day all-cause adjusted mortality**

| **Region** | **Health system** | **MOR** | **Lower CI 95%** | **Upper CI 95%** | **ICC** | **Lower CI 95%** | **Upper CI 95%** |
| --- | --- | --- | --- | --- | --- | --- | --- |
| ON-CAN | CAN | 1.52 | 1.43 | 1.63 | 0.0308 | 0.0198 | 0.0477 |
| ARA-ESP | ESP | 1.35 | 1.18 | 1.58 | 0.0104 | 0.0022 | 0.0465 |
| FIN | FIN | 1.47 | 1.32 | 1.64 | 0.0214 | 0.0095 | 0.0473 |
| SWE | SWE | 1.44 | 1.34 | 1.55 | 0.018 | 0.008 | 0.0402 |
| ALABAMA | USA | 1.36 | 1.26 | 1.46 | 0.0123 | 0.0029 | 0.0501 |
| ARIZONA | USA | 1.39 | 1.29 | 1.51 | 0.0158 | 0.004 | 0.0612 |
| ARKANSAS | USA | 1.4 | 1.26 | 1.55 | 0.0147 | 0.0037 | 0.0572 |
| CALIFORNIA | USA | 1.45 | 1.39 | 1.51 | 0.0227 | 0.0139 | 0.0368 |
| CONNECTICUT | USA | 1.62 | 1.4 | 1.86 | 0.0402 | 0.016 | 0.0976 |
| FLORIDA | USA | 1.47 | 1.4 | 1.56 | 0.0231 | 0.0145 | 0.0367 |
| GEORGIA | USA | 1.47 | 1.36 | 1.58 | 0.0229 | 0.0105 | 0.0493 |
| ILLINOIS | USA | 1.46 | 1.38 | 1.55 | 0.0226 | 0.0115 | 0.0438 |
| INDIANA | USA | 1.48 | 1.38 | 1.6 | 0.0238 | 0.0101 | 0.0549 |
| IOWA | USA | 1.27 | 1.18 | 1.36 | 0.0071 | 0.0005 | 0.0853 |
| KANSAS | USA | 1.7 | 1.48 | 1.98 | 0.0506 | 0.0193 | 0.1261 |
| KENTUCKY | USA | 1.19 | 1.14 | 1.24 | 0.0036 | 0.0001 | 0.1093 |
| LOUISIANA | USA | 1.22 | 1.16 | 1.29 | 0.0048 | 0.0004 | 0.0577 |
| MARYLAND | USA | 1.37 | 1.28 | 1.48 | 0.0136 | 0.004 | 0.0448 |
| MASSACHUSETTS | USA | 1.44 | 1.34 | 1.57 | 0.0202 | 0.0086 | 0.0467 |
| MICHIGAN | USA | 1.39 | 1.31 | 1.47 | 0.0158 | 0.0064 | 0.0386 |
| MINNESOTA | USA | 1.27 | 1.19 | 1.37 | 0.0079 | 0.0006 | 0.0911 |
| MISSISSIPPI | USA | 1.53 | 1.35 | 1.73 | 0.0301 | 0.0094 | 0.0926 |
| MISSOURI | USA | 1.52 | 1.4 | 1.66 | 0.0284 | 0.012 | 0.0657 |
| NEBRASKA | USA | 1.4 | 1.25 | 1.62 | 0.0163 | 0.0022 | 0.1113 |
| NEW JERSEY | USA | 1.17 | 1.13 | 1.21 | 0.0025 | 0.0001 | 0.0518 |
| NEW MEXICO | USA | 1.26 | 1.16 | 1.39 | 0.0099 | 0.0001 | 0.4798 |
| NEW YORK | USA | 1.58 | 1.49 | 1.69 | 0.0361 | 0.0215 | 0.0598 |
| NORTH CAROLINA | USA | 1.49 | 1.39 | 1.6 | 0.0254 | 0.0122 | 0.0522 |
| OHIO | USA | 1.36 | 1.3 | 1.42 | 0.0138 | 0.005 | 0.0375 |
| OREGON | USA | 1.41 | 1.29 | 1.58 | 0.0206 | 0.004 | 0.0997 |
| PENNSYLVANIA | USA | 1.44 | 1.36 | 1.51 | 0.0202 | 0.01 | 0.0403 |
| SOUTH CAROLINA | USA | 1.41 | 1.3 | 1.54 | 0.0175 | 0.0055 | 0.0545 |
| TENNESSEE | USA | 1.42 | 1.32 | 1.53 | 0.0176 | 0.0063 | 0.0482 |
| TEXAS | USA | 1.49 | 1.42 | 1.57 | 0.0264 | 0.0152 | 0.0455 |
| VIRGINIA | USA | 1.39 | 1.31 | 1.49 | 0.0152 | 0.0066 | 0.0348 |
| WASHINGTON | USA | 1.52 | 1.38 | 1.69 | 0.0271 | 0.0112 | 0.0641 |
| WEST VIRGINIA | USA | 1.39 | 1.24 | 1.59 | 0.0165 | 0.0018 | 0.1323 |
| WISCONSIN | USA | 1.78 | 1.58 | 1.99 | 0.0663 | 0.0325 | 0.1303 |

Note: Extreme outliers are excluded: Alaska, Delaware, Nevada, North Dakota, Oklahoma and Rhode Island.

**Table S4.2. 180-day all-cause adjusted mortality**

| **Region** | **Health system** | **MOR** | **Lower CI 95%** | **Upper CI 95%** | **ICC** | **Lower CI 95%** | **Upper CI 95%** |
| --- | --- | --- | --- | --- | --- | --- | --- |
| ON-CAN | CAN | 1.38 | 1.31 | 1.45 | 0.0137 | 0.0088 | 0.0215 |
| ARA-ESP | ESP | 1.16 | 1.08 | 1.26 | 0.0013 | 0.0001 | 0.0241 |
| FIN | FIN | 1.26 | 1.18 | 1.34 | 0.0037 | 0.0011 | 0.0119 |
| SWE | SWE | 1.28 | 1.22 | 1.36 | 0.0055 | 0.0019 | 0.016 |
| ALABAMA | USA | 1.3 | 1.23 | 1.39 | 0.0066 | 0.002 | 0.0214 |
| ARIZONA | USA | 1.18 | 1.14 | 1.23 | 0.0023 | 0.0001 | 0.0408 |
| ARKANSAS | USA | 1.35 | 1.23 | 1.49 | 0.0094 | 0.0029 | 0.0302 |
| CALIFORNIA | USA | 1.4 | 1.35 | 1.45 | 0.0145 | 0.0097 | 0.0215 |
| CONNECTICUT | USA | 1.4 | 1.27 | 1.55 | 0.0133 | 0.0046 | 0.0375 |
| DELAWARE | USA | 1.53 | 1.14 | 2.24 | 0.0253 | 0.0031 | 0.1805 |
| FLORIDA | USA | 1.41 | 1.35 | 1.48 | 0.0148 | 0.0097 | 0.0225 |
| GEORGIA | USA | 1.34 | 1.28 | 1.43 | 0.0097 | 0.0042 | 0.022 |
| ILLINOIS | USA | 1.35 | 1.29 | 1.41 | 0.0102 | 0.0054 | 0.0194 |
| INDIANA | USA | 1.26 | 1.21 | 1.32 | 0.0053 | 0.0015 | 0.018 |
| IOWA | USA | 1.03 | 1.02 | 1.04 | 0.0001 | 0 | 1 |
| KANSAS | USA | 1.33 | 1.22 | 1.46 | 0.0088 | 0.0016 | 0.0476 |
| KENTUCKY | USA | 1.22 | 1.17 | 1.28 | 0.0037 | 0.0006 | 0.0232 |
| LOUISIANA | USA | 1.33 | 1.24 | 1.44 | 0.0088 | 0.0029 | 0.0268 |
| MARYLAND | USA | 1.28 | 1.21 | 1.36 | 0.0054 | 0.0012 | 0.0238 |
| MASSACHUSETTS | USA | 1.33 | 1.25 | 1.41 | 0.0084 | 0.0032 | 0.0218 |
| MICHIGAN | USA | 1.37 | 1.29 | 1.45 | 0.0116 | 0.0056 | 0.0239 |
| MINNESOTA | USA | 1.29 | 1.2 | 1.4 | 0.007 | 0.0015 | 0.0317 |
| MISSISSIPPI | USA | 1.28 | 1.19 | 1.38 | 0.006 | 0.0011 | 0.0317 |
| MISSOURI | USA | 1.41 | 1.31 | 1.51 | 0.0144 | 0.0063 | 0.0328 |
| NEBRASKA | USA | 1.39 | 1.22 | 1.6 | 0.0127 | 0.0031 | 0.0511 |
| NEW JERSEY | USA | 1.35 | 1.27 | 1.44 | 0.0096 | 0.004 | 0.0226 |
| NEW MEXICO | USA | 1.43 | 1.27 | 1.65 | 0.0179 | 0.0038 | 0.0793 |
| NEW YORK | USA | 1.4 | 1.34 | 1.48 | 0.0141 | 0.0085 | 0.0233 |
| NORTH CAROLINA | USA | 1.37 | 1.29 | 1.44 | 0.0106 | 0.005 | 0.022 |
| NORTH DAKOTA | USA | 1.17 | 1.06 | 1.34 | 0.002 | 0 | 0.9866 |
| OHIO | USA | 1.2 | 1.17 | 1.24 | 0.0031 | 0.0006 | 0.0161 |
| OKLAHOMA | USA | 1.23 | 1.17 | 1.29 | 0.0035 | 0.0006 | 0.0213 |
| OREGON | USA | 1.12 | 1.09 | 1.17 | 0.0013 | 0 | 0.78 |
| PENNSYLVANIA | USA | 1.36 | 1.3 | 1.43 | 0.0111 | 0.0059 | 0.0207 |
| RHODE ISLAND | USA | 1.55 | 1.24 | 2.03 | 0.0297 | 0.0071 | 0.1169 |
| SOUTH CAROLINA | USA | 1.36 | 1.26 | 1.46 | 0.0102 | 0.0036 | 0.0283 |
| TENNESSEE | USA | 1.21 | 1.16 | 1.26 | 0.0029 | 0.0005 | 0.0152 |
| TEXAS | USA | 1.39 | 1.33 | 1.45 | 0.0129 | 0.008 | 0.021 |
| VIRGINIA | USA | 1.39 | 1.31 | 1.48 | 0.0127 | 0.0064 | 0.0248 |
| WASHINGTON | USA | 1.48 | 1.36 | 1.63 | 0.0206 | 0.01 | 0.0419 |
| WEST VIRGINIA | USA | 1.39 | 1.24 | 1.57 | 0.0128 | 0.0033 | 0.0484 |
| WISCONSIN | USA | 1.38 | 1.3 | 1.49 | 0.0141 | 0.0051 | 0.0386 |

Note: Extreme outliers are excluded: Alaska and Nevada.

**Table S5. Median predicted mortality rates by hospital by quintile of hip fracture surgery volume**

|  | **30-day adjusted mortality rate (per 1,000 hip repairs)** | | **180-day adjusted mortality rate [range] (per 1,000 hip repairs)** | |
| --- | --- | --- | --- | --- |
| **Quintile (surgery episodes)** | **P50 [Range Min-Max]** | **Dif (Ratio Max/Min)** | **P50 [Range Min-Max]** | **Dif (Ratio Max/Min)** |
| 1 (<= 89) | 39.9 [13.7-97] | 83.3 (7.08) | 134.7  [64.1-243.9] | 179.8 (3.8) |
| 2 between (90-142) | 40.8 [9.5-90] | 80.5 (9.47) | 138.9  [72.7-222.5] | 149.8 (3.06) |
| 3 between (143-215) | 40.7 [21.1-87.8] | 66.7 (4.16) | 135.2  [72.4-214.6] | 142.2 (2.96) |
| 4 between (216-343) | 40.2 [21.0-83.6] | 62.6 (3.98) | 136.2  [70.8-215.1] | 144.3 (3.04) |
| 5 (>= 344) | 40.7 [21.7-74.1] | 52.4 (3.41) | 136 [76.1-199.3] | 123.2 (2.62) |

**Figure S1. CONSORT chart per participant health system**

| 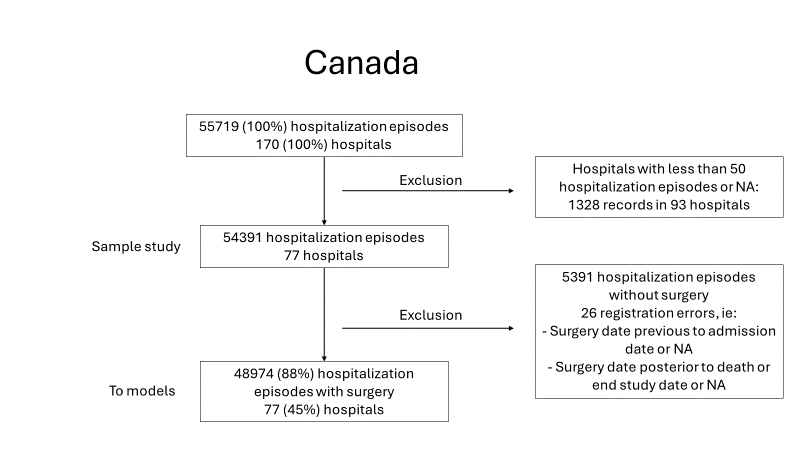 | 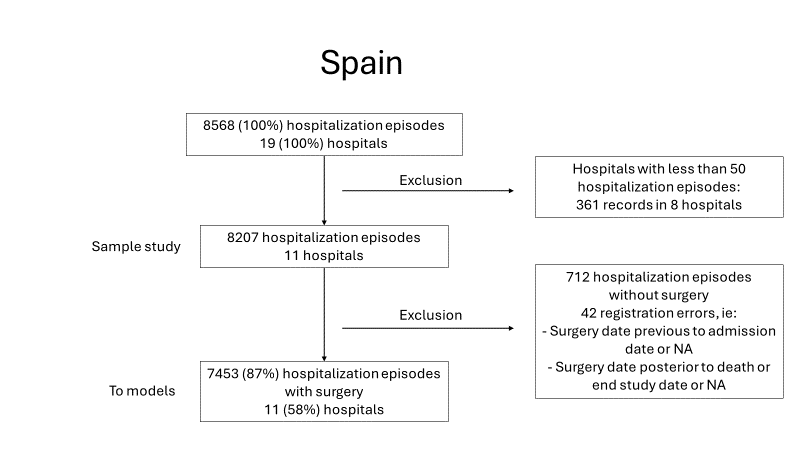 |
| --- | --- |
| 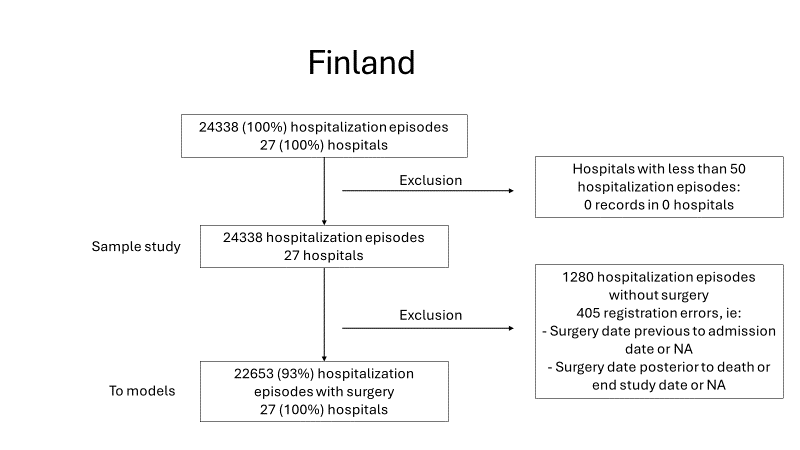 | 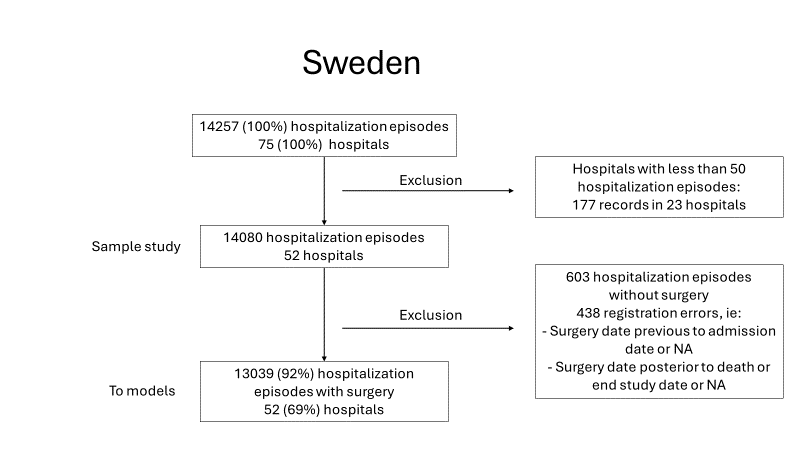 |

| 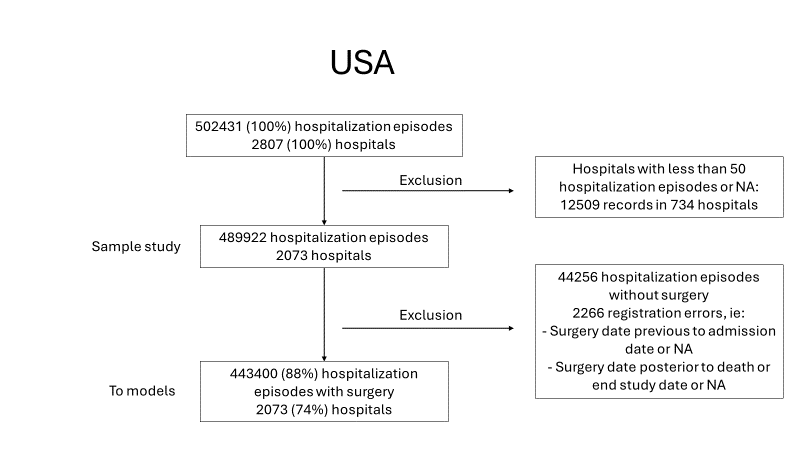 |
| --- |

**Figure S2. GAMM models: Distribution of ORs**

| **S2.1. - Risk of death at 30 days** | **S2.2. - Risk of death at 180 days** |
| --- | --- |
| **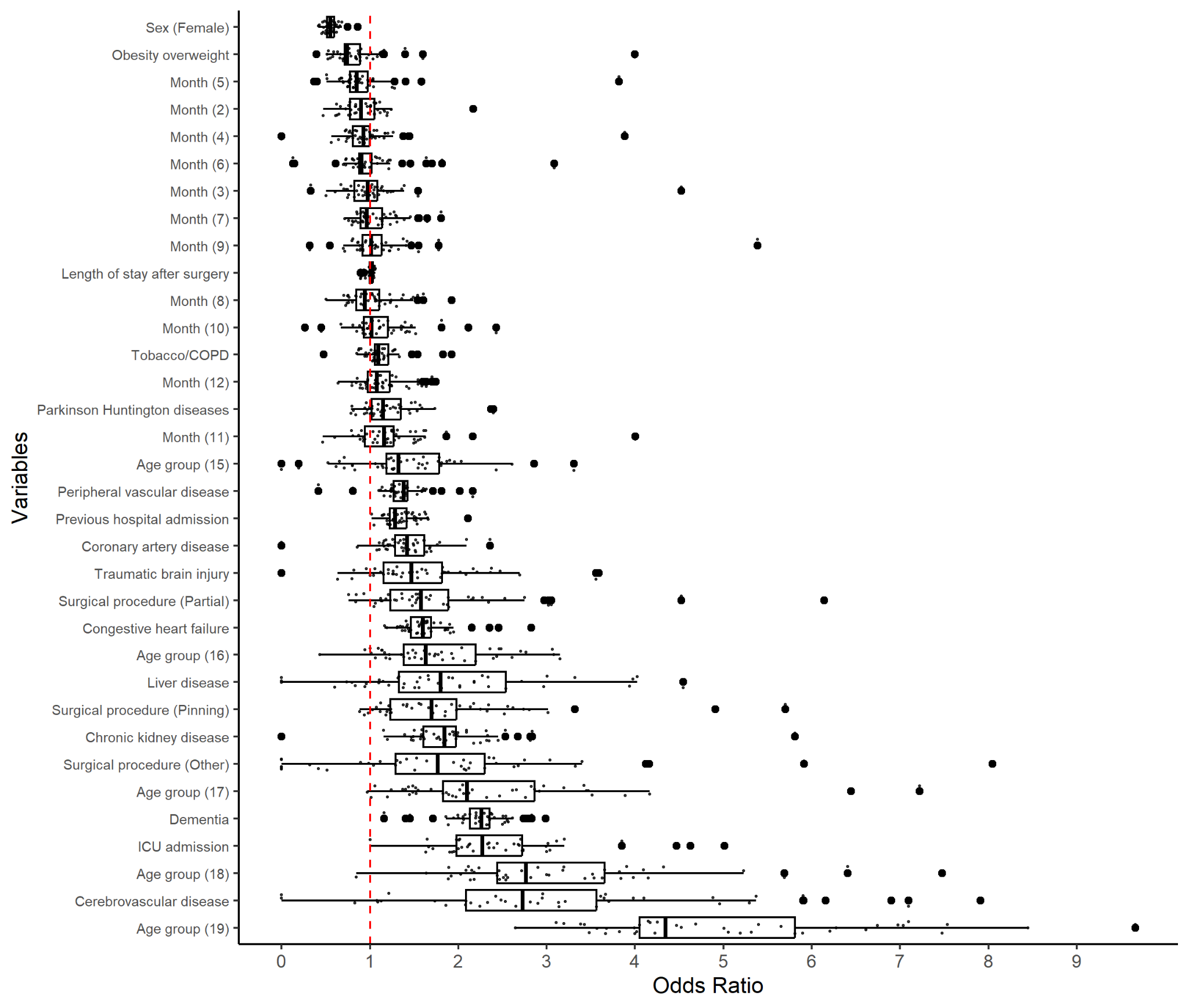** | **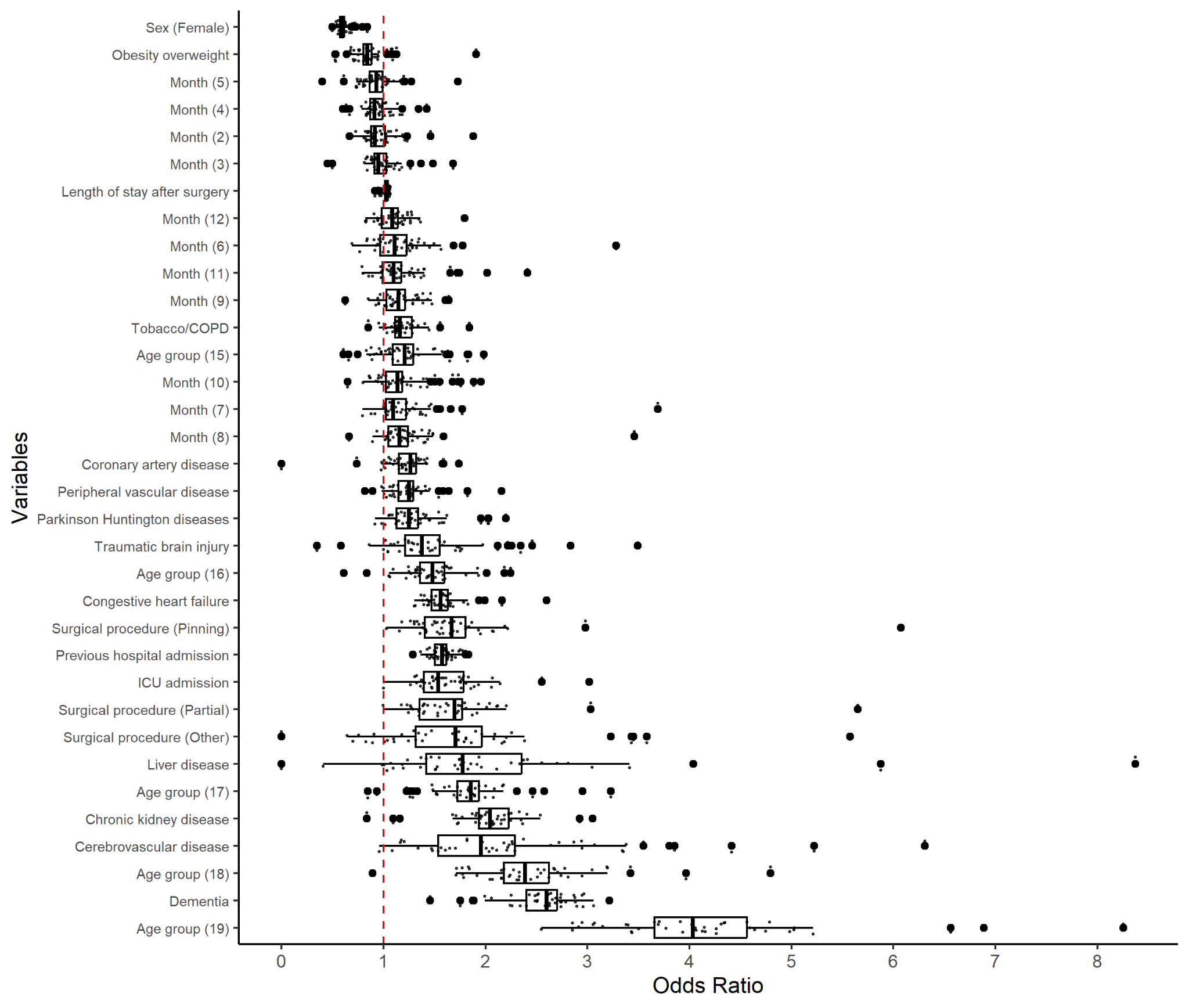** |
| **Legend**. The distribution of Odds ratios by variable as produced by the local GAMM models for each region are shown as box plots. Each dot is the point estimate (Odds ratio) for each variable in a particular region. The vertical red dashed line marks an Odds ratio of 1. Extreme outliers are excluded - Figure S2.1: 0.4% data was removed. Figure S2.2: 0.06% data was removed. | |

**Figure S3. GAMM models: Distribution of residuals**

| **S3.1. - Model: 30-day all-cause mortality** | **S3.2. - Model: 180-day all-cause mortality** |
| --- | --- |
| **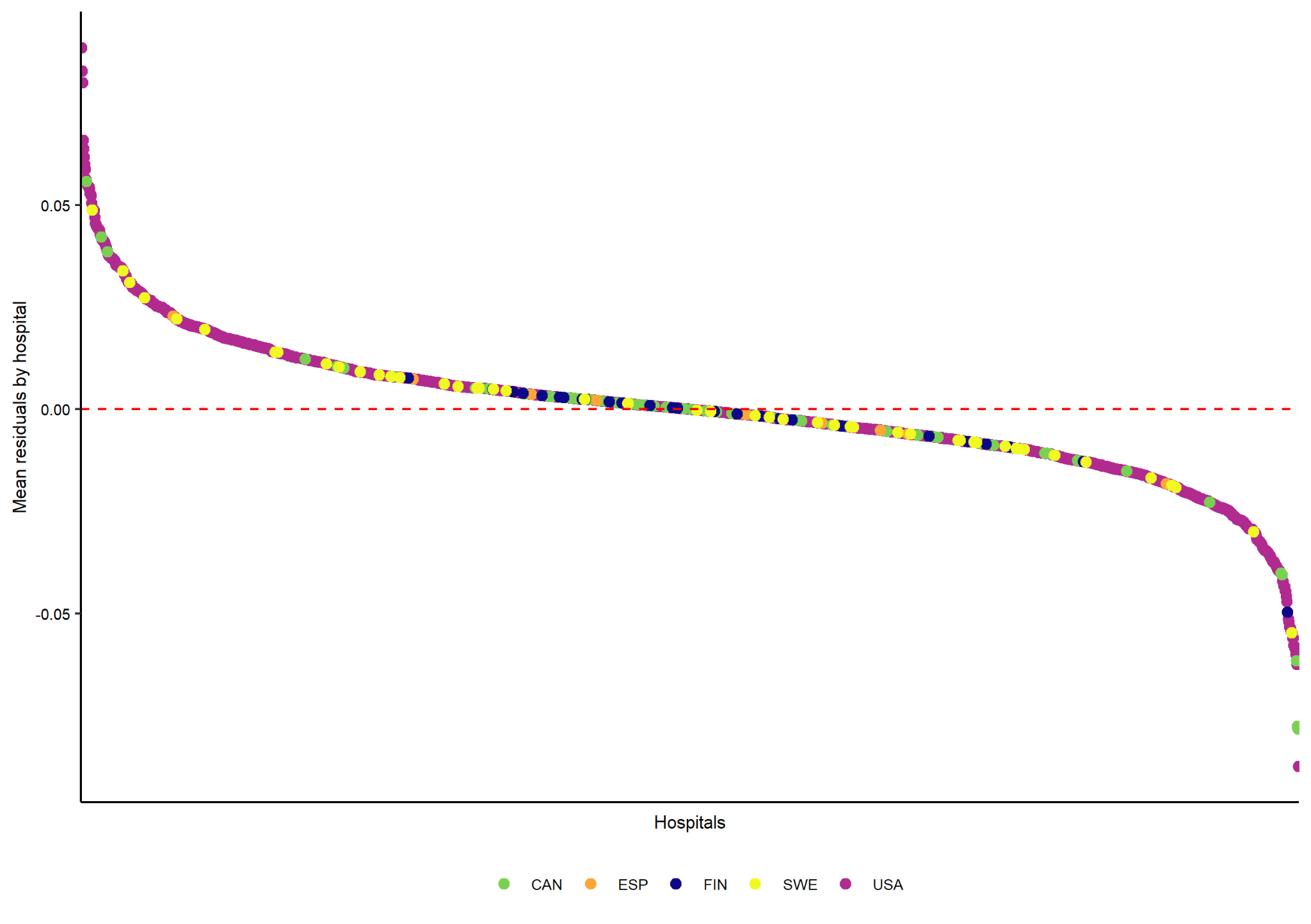** | **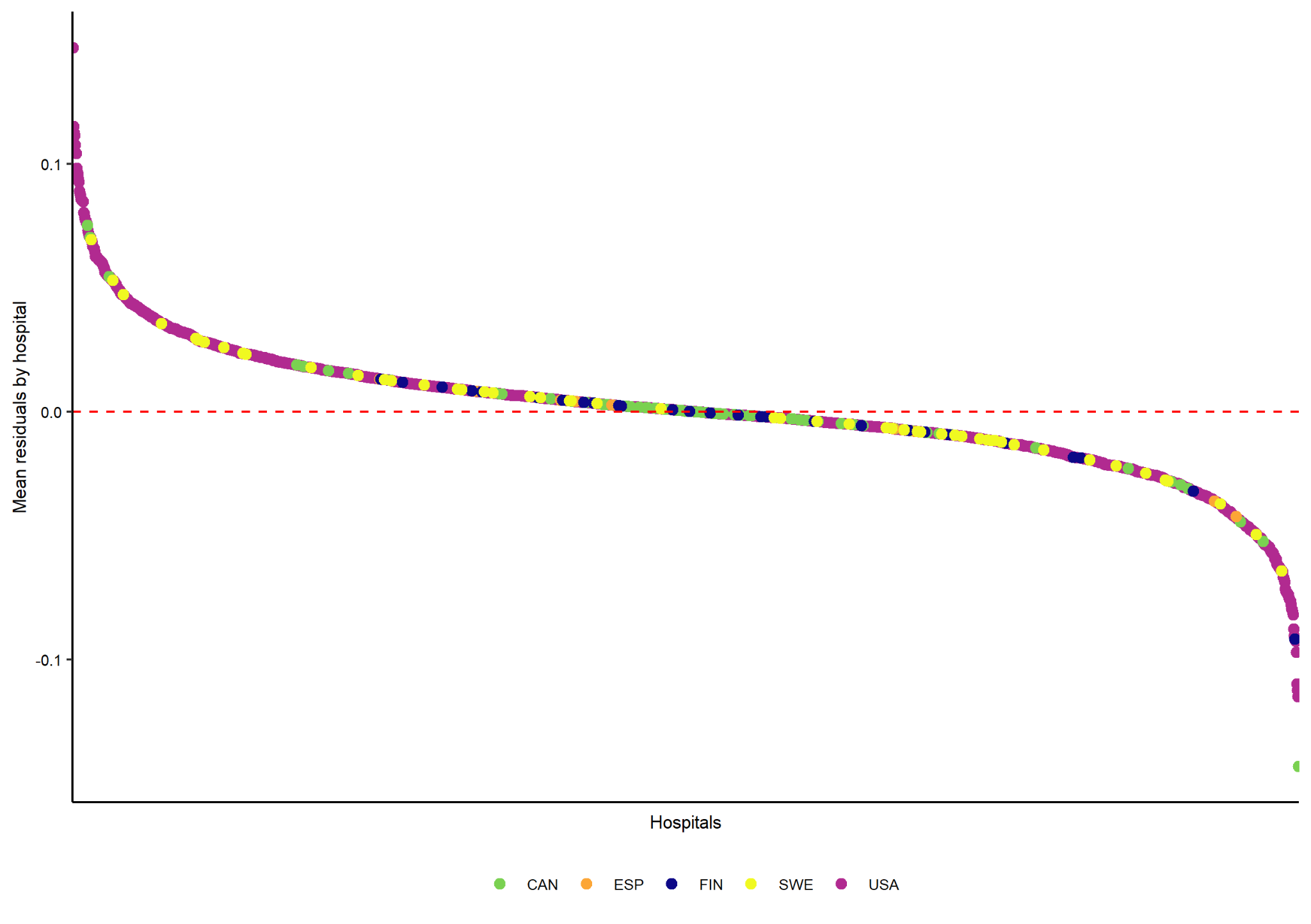** |
| **Legend.** Distribution of mean residuals by hospital. Each dot represents the mean residual for each hospital and is coloured by the health system. The GAMM residuals are expected to follow a normal distribution with a mean of 0, marked with the horizontal red dashed line. | |

**Figure S4. Distribution (by hospital) of the number of comorbidities per hospitalisation episode**

| **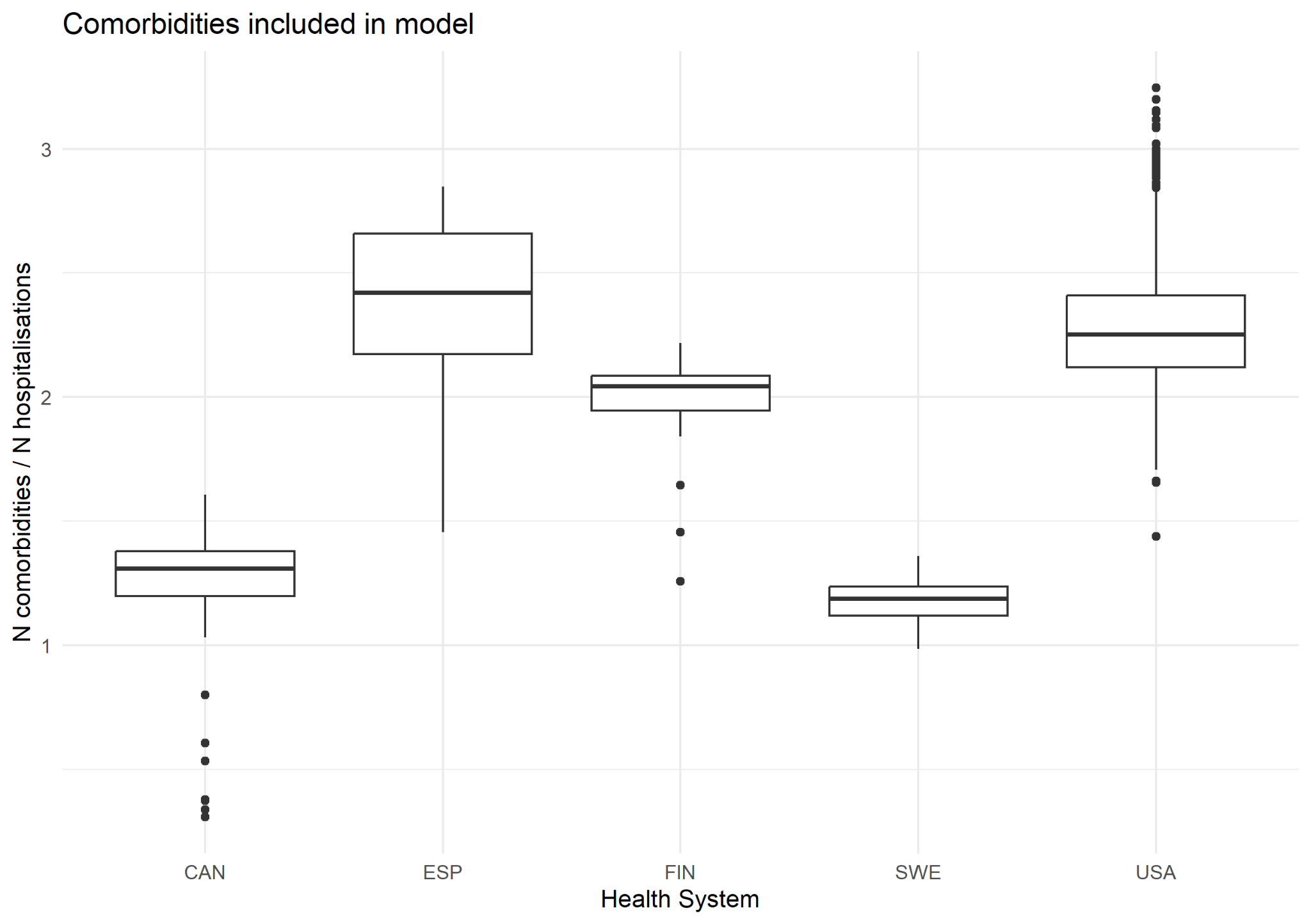** |
| --- |
| **Legend.** Distribution of the number of comorbidities (included in model) per hospitalisation episode. Each boxplot represents the distribution of ratios (N comorbidities / N hospitalisations) where each point of the distribution is the ratio for a hospital belonging to its corresponding health system. |

**Figure S5. Median predicted mortality rates vs. number of comorbidities per hospitalisation episode**

| **S5.1. - 30-day all-cause predicted mortality rates** | **S5.2. - 180-day all-cause predicted mortality rates** |
| --- | --- |
| **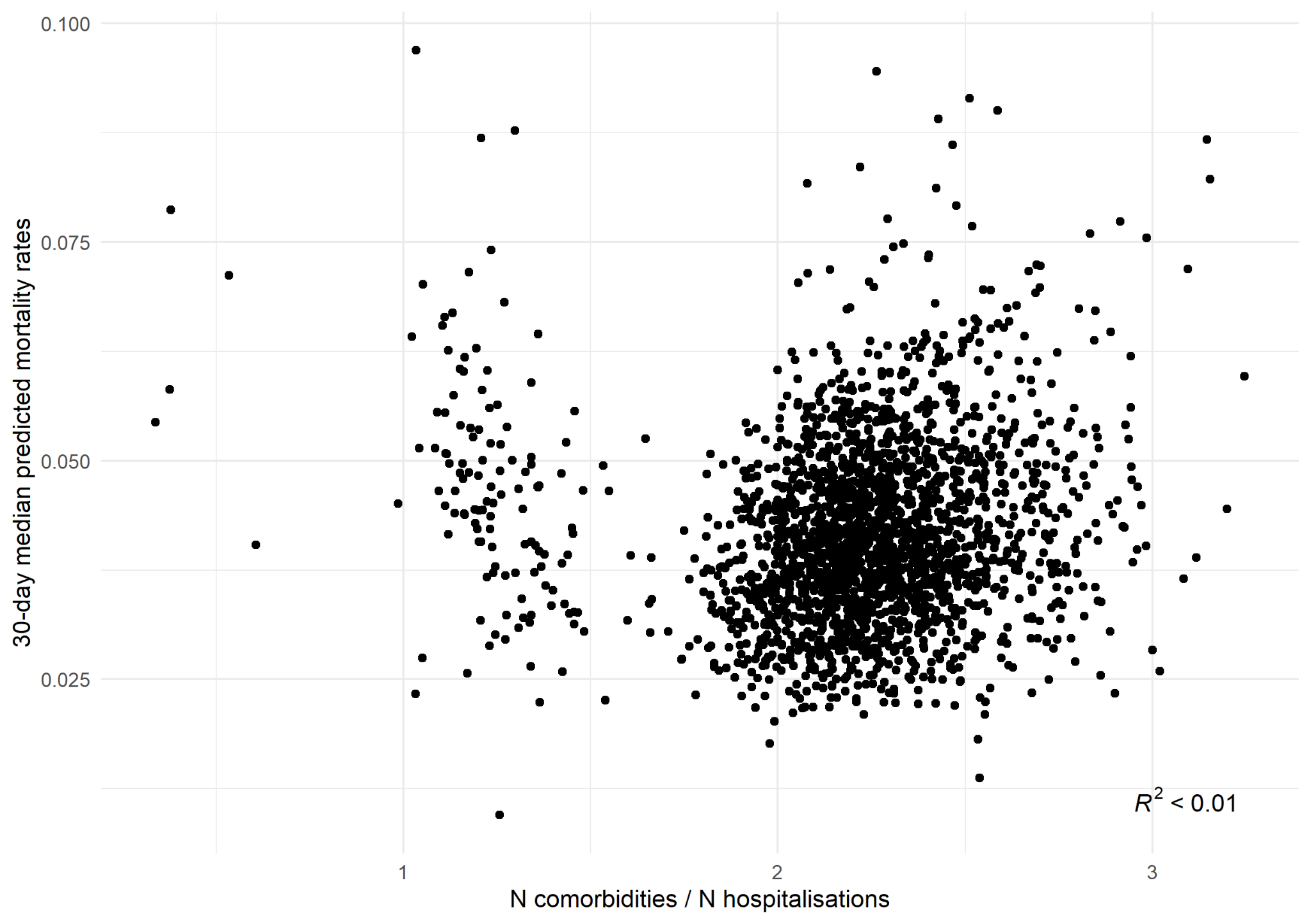** | **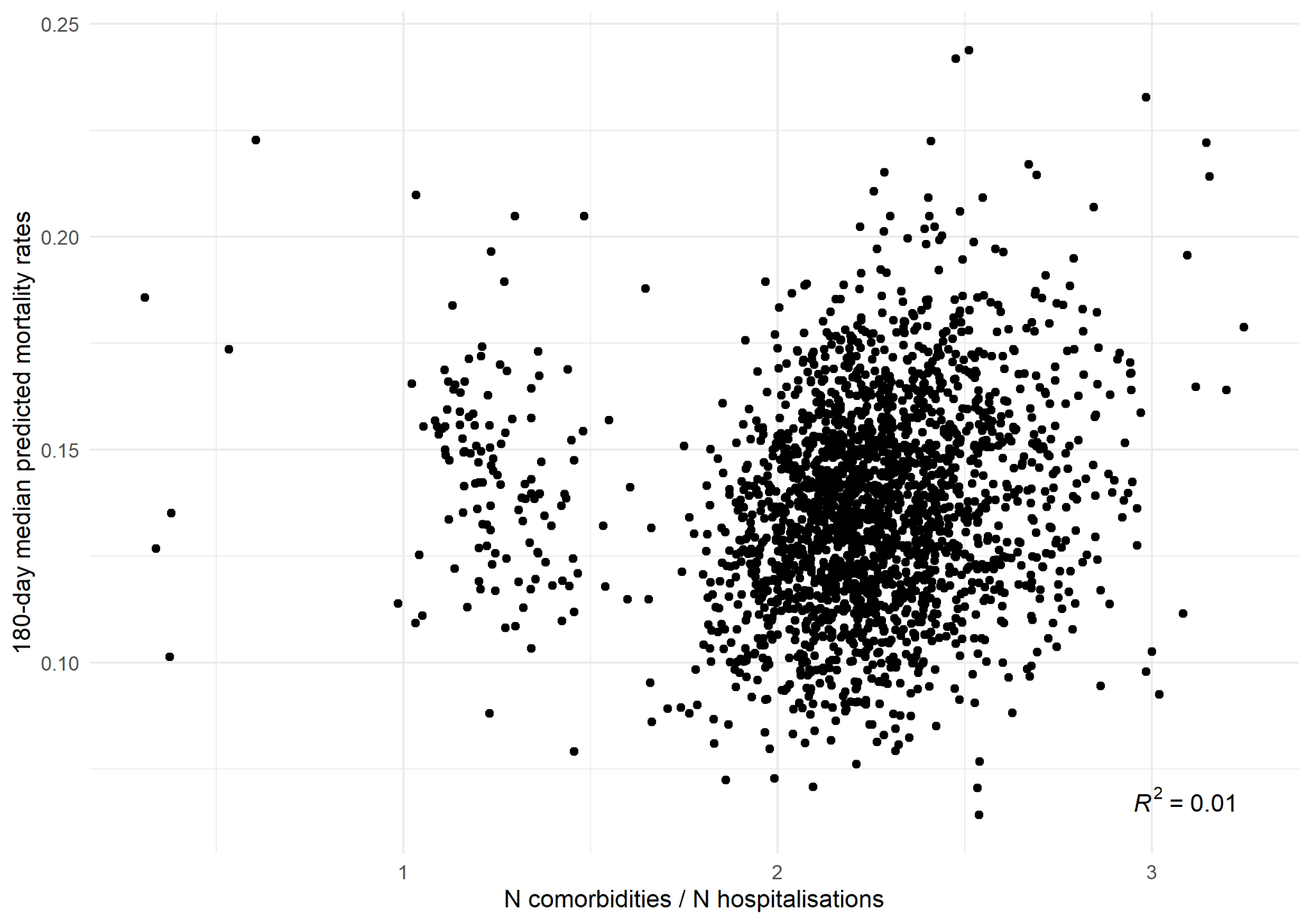** |
| **Legend**. Median predicted mortality rates versus the number of comorbidities per hospitalization episode. Each dot represents a hospital. The figure demonstrates that there is no significant association (r² = 0.01) between the number of recorded comorbidities and the adjusted mortality rates at 30 and 180 days. | |

**Mathematical Notation**

1. **GAMM models**

**1.1 30-day all-cause mortality**

$$exitus 30 days bl_{ij}\sim Binomial(n=1,prob_{exitus 30 days bl_{ij}=1}=\hat{P})$$

$$log[\frac{\hat{P}}{1-\hat{P}}]=\beta_{0}+f_{1}(futime adm surg_{ij}) + \beta_{1}(sex cd_{female_{ij}}) + \beta_{2}(surgical procedure cd_{partial_{ij}})+$$

$$\beta_{3}(surgical procedure cd_{pinning_{ij}})+ \beta_{4}(surgical procedure cd_{other_{ij}})+ \beta_{5}(icu admission bl_{ij}) +$$

$$\beta_{6}(chronic kidney disease bl_{ij})+ \beta_{7}(tobacco/copd bl_{ij})+ \beta_{8}(obesity/overweight bl_{ij}) +$$

$$\beta_{9}(dementia bl_{ij})+ \beta_{10}(liver disease bl_{ij})+ \beta_{11}(parkinson/huntington diseases bl_{ij}) +$$

$$\beta_{12}(congestive heart failure bl_{ij})+ \beta_{13}(coronary artery disease bl_{ij})+$$

$$\beta_{14}(cerebrovascular disease bl_{ij}) + \beta_{15}(peripheral vascular disease bl_{ij}) +$$

$$\beta_{16}(traumatic brain injury bl_{ij}) + \beta_{17}(month_{2_{ij}}) + \beta_{18}(month_{3_{ij}}) + \beta_{19}(month_{4_{ij}}) + \beta_{20}(month_{5_{ij}}) +$$

$$\beta_{21}(month_{6_{ij}}) + \beta_{22}(month_{7_{ij}}) + \beta_{23}(month_{8_{ij}}) + \beta_{24}(month_{9_{ij}}) + \beta_{25}(month_{{10}_{ij}}) + \beta_{26}(month_{{11}_{ij}}) +$$

$$\beta_{27}(month_{{12}_{ij}}) + \beta_{28}({age group}_{{15}_{ij}}) + \beta_{29}({age group}_{{16}_{ij}}) + \beta_{30}({age group}_{{17}_{ij}}) +$$

$$\beta_{31}({age group}_{{18}_{ij}}) + \beta_{32}({age group}_{{19}_{ij}}) + \beta_{33}(previous hospital admission bl_{ij}) +$$

$$\beta_{34}(length of stay after surgery_{ij}) + \alpha_{j}$$

$$\alpha_{j}\sim N(\mu_{\alpha},\sigma_{\alpha}^{2}), for hospital st j=1,...,J;$$

**1.2 180-day all-cause mortality**

$$exitus 180 days bl_{ij}\sim Binomial(n=1,prob_{exitus 180 days bl_{ij}=1}=\hat{P})$$

$$log[\frac{\hat{P}}{1-\hat{P}}]=\beta_{0}+f_{1}(futime adm surg_{ij}) + \beta_{1}(sex cd_{female_{ij}}) + \beta_{2}(surgical procedure cd_{partial_{ij}})+$$

$$\beta_{3}(surgical procedure cd_{pinning_{ij}})+ \beta_{4}(surgical procedure cd_{other_{ij}})+ \beta_{5}(icu admission bl_{ij}) +$$

$$\beta_{6}(chronic kidney disease bl_{ij})+ \beta_{7}(tobacco/copd bl_{ij})+ \beta_{8}(obesity/overweight bl_{ij}) +$$

$$\beta_{9}(dementia bl_{ij})+ \beta_{10}(liver disease bl_{ij})+ \beta_{11}(parkinson/huntington diseases bl_{ij}) +$$

$$\beta_{12}(congestive heart failure bl_{ij})+ \beta_{13}(coronary artery disease bl_{ij})+$$

$$\beta_{14}(cerebrovascular disease bl_{ij}) + \beta_{15}(peripheral vascular disease bl_{ij}) +$$

$$\beta_{16}(traumatic brain injury bl_{ij}) + \beta_{17}(month_{2_{ij}}) + \beta_{18}(month_{3_{ij}}) + \beta_{19}(month_{4_{ij}}) + \beta_{20}(month_{5_{ij}}) +$$

$$\beta_{21}(month_{6_{ij}}) + \beta_{22}(month_{7_{ij}}) + \beta_{23}(month_{8_{ij}}) + \beta_{24}(month_{9_{ij}}) + \beta_{25}(month_{{10}_{ij}}) + \beta_{26}(month_{{11}_{ij}}) +$$

$$\beta_{27}(month_{{12}_{ij}}) + \beta_{28}({age group}_{{15}_{ij}}) + \beta_{29}({age group}_{{16}_{ij}}) + \beta_{30}({age group}_{{17}_{ij}}) +$$

$$\beta_{31}({age group}_{{18}_{ij}}) + \beta_{32}({age group}_{{19}_{ij}}) + \beta_{33}(previous hospital admission bl_{ij}) +$$

$$\beta_{34}(length of stay after surgery_{ij}) + \alpha_{j}$$

$$\alpha_{j}\sim N(\mu_{\alpha},\sigma_{\alpha}^{2}), for hospital st j=1,...,J;$$

1. **Median Odds Ratio (MOR) equation[15]**

$$MOR = exp(\sqrt{2\times\sigma_{\alpha}^{2}}\times0.6745)$$

In our case, ‘${\sigma_{\alpha}^{2}}$’ is the hospital variance.

1. **Whiskers (Box-Plot) equation[22]**

$$Upper whisker = min(max(x),Q_{3} + 1.5 \times IQR); Lower whisker =max(min(x),Q_{1} - 1.5 \times IQR)$$

In our case, ‘x’ is the odds ratio of each region for each variable.
